# Planktonic and Sessile Artificial Colonic Microbiota Harbor Distinct Composition and Reestablish Differently upon Frozen and Freeze-Dried Long-Term Storage

**DOI:** 10.1128/mSystems.00521-19

**Published:** 2020-01-21

**Authors:** Lea Bircher, Clarissa Schwab, Annelies Geirnaert, Anna Greppi, Christophe Lacroix

**Affiliations:** a Laboratory of Food Biotechnology, Institute of Food, Nutrition and Health, ETH Zürich, Zurich, Switzerland; Luxembourg Centre for Systems Biomedicine

**Keywords:** FMT, bacterial lifestyle, colonic microbiota, cryopreservation, lyophilization

## Abstract

Biofilm-associated, sessile communities represent the major bacterial lifestyle, whereas planktonic cells mainly appear during initial colonization of new surfaces. Previous research, mainly performed with pathogens, demonstrated increased environmental stress tolerance of biofilm-growing compared to planktonic bacteria. The lifestyle-specific stress response of colonic microbiota, both natural and fermentation produced, has not been addressed before. Planktonic and sessile “artificial” colonic microbiota delivered by PolyFermS continuous fermentation models can provide a controllable and reproducible alternative to fecal transplantation in treating gastrointestinal disorders. We therefore characterized planktonic and sessile microbiota produced in two PolyFermS models inoculated with immobilized fecal microbiota and comparatively tested their levels of tolerance of frozen storage (–80°C) and freeze-dried storage (4°C) for 9 months to mimic preservation strategies for therapeutic applications. Sessile microbiota harbored next to shared taxa a unique community distinguishable from planktonic microbiota. *Synergistetes* and *Proteobacteria* were highly represented in sessile microbiota, while *Firmicutes* were more abundant in planktonic microbiota. The community structure and metabolic activity of both microbiota, monitored during standardized reactivation batch fermentations, were better preserved after frozen storage than dried storage, indicated by higher Bray-Curtis similarity and enhanced recovery of metabolite production. For both lifestyles, reestablishment of *Bacteroidaceae* was impaired after frozen and dried storage along with reduced propionate formation. In contrast, butyrate production was maintained after reactivation despite compositional rearrangements within the butyrate-producing community. Unexpectedly, the rate of recovery of metabolite production was lower after preservation of sessile than planktonic microbiota. We speculate that higher functional dependencies between microbes might have led to the lower stress tolerance of sessile than planktonic microbiota.

**IMPORTANCE** Fecal microbiota transplantation has been successfully applied in the treatment of recurrent Clostridium difficile infection and has been suggested as an alternative therapy for other intestinal disorders such as inflammatory bowel disease or metabolic syndrome. “Artificial” colonic microbiota delivered by PolyFermS continuous fermentation models can provide a controllable and reproducible alternative to fecal transplantation, but effective preservation strategies must be developed. In this study, we systematically investigated the response of sessile and planktonic artificial colonic microbiota to cryopreservation and lyophilization. We suggest that functional redundancy is an important factor in providing functional stability with respect to exposure to stress during processing and storage. Functional redundancy in compositionally reduced microbial systems may be considered when designing microbial products for therapy.

## Abstract (precis)

Fecal microbiota transplantation has been successfully applied in the treatment of recurrent Clostridium difficile infection and has been suggested as an alternative therapy for other intestinal disorders such as inflammatory bowel disease or metabolic syndrome. “Artificial” colonic microbiota delivered by PolyFermS continuous fermentation models can provide a controllable and reproducible alternative to fecal transplantation, but effective preservation strategies must be developed. In this study, we systematically investigated the response of sessile and planktonic artificial colonic microbiota to cryopreservation and lyophilization. We suggest that functional redundancy is an important factor in providing functional stability with respect to exposure to stress during processing and storage. Functional redundancy in compositionally reduced microbial systems may be considered when designing microbial products for therapy.

## INTRODUCTION

The human gastrointestinal tract is a densely populated ecosystem that harbors more than 10^14^ microorganisms composed of more than 1,000 different species at a concentration excessing 10^10^ bacterial cells per gram content (1, 2). Several studies have reported the occurrence of biofilm-like structures in the colonic habitat, especially on food particles and attached to the mucus layer (3–5). Microscopic observations demonstrated the appearance of interspecies microcolonies and bacterial biofilms on the colonic mucosa in the large intestine and on plant particles originating from fecal matter (6). The microbial composition of particle-associated communities extracted from fecal samples differed from the composition in the suspended fraction; higher abundances of representatives of the phylum *Firmicutes* were associated with insoluble particles whereas *Bacteroidetes* dominated in the liquid phase (7). Likewise, the compositions of biofilms on mucin surfaces were different *in vitro* (8) and *in vivo* (9) from those of luminal or planktonic counterparts, indicating that microbial communities of different lifestyles coexist in the gastrointestinal tract.

The PolyFermS continuous *in vitro* intestinal fermentation model, inoculated with fecal microbiota immobilized in highly porous polysaccharide gel beads, was designed to represent both the planktonic and biofilm-associated lifestyles of complex human colonic microbiota over an extended test period of 150 days (10, 11). The PolyFermS model allows researchers to maintain and cultivate the distinct microbial profile of the fecal consortia used for inoculation (12). Entrapping fecal microbiota in gel beads enables growth of sessile bacteria within the bead structure and overcomes major restrictions of other intestinal fermentation models that can reproduce only the free-cell microbes (10). Cells continuously released from the beads in the bulk medium contribute to the seeding and growth of the planktonic community. The use of “fecal beads” was shown to stabilize the modeled planktonic community, preventing washout of less-competitive bacteria and enabling stable operation of the continuous model for several months (13–15). Immobilization in porous polymer beads was suggested as a model for “artificial” biofilms, based on specific expression patterns in immobilized microbiota, cell and physiochemical gradient formation in the beads, and active detachment of cells in the surrounding liquid part, as observed in “authentic” biofilms (16). Converting from the sessile lifestyle to the planktonic lifestyle and vice versa involves changes in gene expression and physiological modifications of the alternating cells (16). For example, genes linked to stress response, cell envelope function, and iron-sulfur metabolism are upregulated in biofilms compared to planktonic communities (17). Sessile bacteria may have an advantage over planktonic bacteria due to higher environmental stress tolerance of biofilm-grown cells, physical stress protection provided by the matrix, nutrient capture that is enhanced by the spongy structure of the exopolymeric matrix, and the increased enzyme retention that provides more-efficient substrate conversion (18). Various studies reported increased environmental stress tolerance of biofilms formed by single or mixed bacterial species compared to planktonic cultures (19–24). Investigations included species from both Gram-positive and Gram-negative taxa and a variety of stressors such as disinfectants, antibiotics, bile salts, acids, and solvents. To our knowledge, lifestyle-dependent stress resistance of complex intestinal microbial communities has never been investigated in spite of the increasing interest in artificial colonic microbiota as a replacement of fecal microbiota transplantation in the treatment of gastrointestinal disorders (25–28).

Applying human feces-derived microbiota in therapy demands production and preservation technologies that warrant the composition and functionality of microbial-based products but are potentially harmful to bacterial cells (28). Cryopreservation and lyophilization are the most commonly applied bacterial preservation techniques used in culture collections as well as industry (29). Both preservation methods involve mechanical and osmotic stressors that can be lethal to bacterial cells. Intra- and extracellular ice crystals formed during the freezing process can directly rupture cellular membranes or indirectly affect cells due to enhanced osmotic pressure generated by solute concentrations in the remaining unfrozen fractions (30, 31). Preservation by lyophilization also demands an initial freezing step; thus, the bacterial cells are exposed to the same stressors as in cryopreservation. In addition, the gradual removal of water further increases osmotic pressure and can cause severe drying-related injuries to the cell wall and to cellular components (32). On the basis of the enhanced stress tolerance of biofilm-associated bacteria, we hypothesized that sessile microbiota (sessM) in beads derived from the PolyFermS bioreactor is more resistant to stressors induced by preservation processes than planktonic microbiota (plankM).

In this work, we comparatively investigated the composition and activity of sessM and plankM deriving from the PolyFermS model, mimicking the adult proximal colon, by 16S rRNA gene amplicon sequencing, quantitative real-time PCR (qPCR), and high-pressure liquid chromatography-refractive index detection (HPLC-RI). We tested the lifestyle-specific response upon the application of two preservation methods most commonly used for bacterial preparations, namely, freezing and lyophilization. A storage period of 9 months was selected to represent the conditions suggested for storing fecal material for fecal microbiota transplantation (FMT) (33). To improve preservation survival, the protectants sucrose and inulin (both 5% [wt/vol]) and combinations of sucrose and inulin (both 5% [wt/vol]) and glycerol (15% [vol/vol]), previously developed for stabilizing strict anaerobic gut microbes, were added for lyophilization and cryopreservation, respectively (34). Composition and metabolic activities of preserved plankM and sessM were evaluated in small-scale, strictly anaerobic batch cultures over 24 h, and the results were compared to those seen with batch cultures inoculated with fresh microbiota.

## RESULTS AND DISCUSSION

### Lifestyle of artificial colonic microbiota impacts bacterial community composition and metabolic activity.

**FIG 1:**
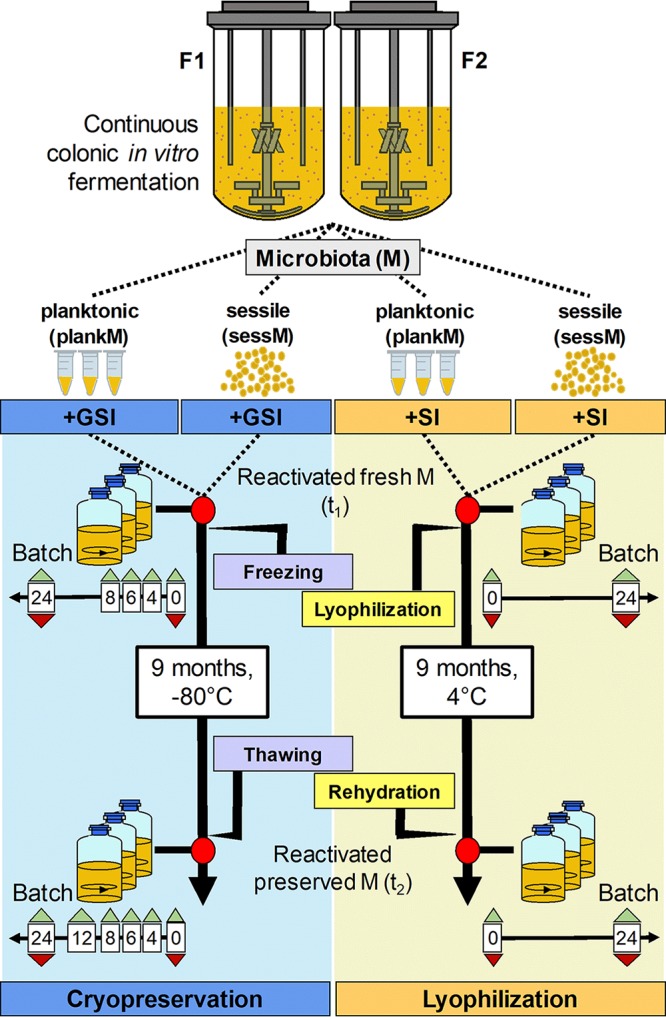
Preservation experiments. Planktonic artificial colonic microbiota (plankM) and sessile artificial colonic microbiota (sessM) were obtained from fermentation F1 and F2. Microbiotas were reactivated in batch fermentation at two different time points (red dots). The first batch fermentation was performed with fresh microbiota after incubation with protective buffers containing sucrose and inulin (SI) or glycerol, sucrose, and inulin (GSI) (t_1_). The second batch fermentation was conducted with the preserved microbiota after freezing at –80°C, lyophilization, and storage for 9 months at 4°C (t_2_). Samples were collected from batch fermentations at different incubation time points for microbiota profiling (red triangles) and metabolite analysis (green triangles) in triplicate.

Artificial colonic microbiota were produced with two independent immobilized cell continuous intestinal models mimicking the conditions of the adult proximal colon (F1 and F2), inoculated with immobilized fecal microbiota in polysaccharide gel beads (30% [vol/vol]) from two healthy donors. Both fermentations maintained stable metabolic profiles of main short-chain fatty acids (SCFA), acetate, propionate, and butyrate during the overall test periods of 48 and 19 days of continuous fermentation, respectively (see Fig. S1 in the supplemental material). Each fermentation system cultivated colonic microbiota of two different lifestyles: microbiota in the free cell state, here referred to as planktonic microbiota (plankM), or microbiota entrapped in 1-to-2-mm-diameter gellan-xanthan beads, here referred to as sessile microbiota (sessM) (Fig. 1). The composition of plankM and sessM of both models was analyzed by 16S rRNA gene amplicon sequencing of the V4 variable region (for the sampling scheme, see Fig. S1) and compared semiquantitatively with that of the fecal microbiota used to inoculate the models. The plankM population was harvested twice on different fermentation days: first for cryopreservation (plankM_F1.1 and plankM_F2.1) and then for lyophilization (plankM_F1.2 and plankM_F2.2). The sessM population was harvested once, at the end of the fermentation experiments. The main metabolites from the carbohydrate and protein fermentations in the effluent of the PolyFermS model were determined by HPLC-RI.

10.1128/mSystems.00521-19.1 FIG S1 Metabolic activity of fermentation systems. Absolute metabolite concentrations in fermentation 1 (A) and fermentation 2 (B) were measured by HPLC-RI. Days of sampling of sessM and plankM for preservation trials are marked with red arrows. PlankM_F1.1 and plankM_F2.1 were sampled for the cryopreservation assay and plankM_F1.2 and plankM_F2.2 for the lyophilization trial. Download FIG S1, TIF file, 0.2 MB. Copyright © 2020 Bircher et al. 2020 Bircher et al. This content is distributed under the terms of the Creative Commons Attribution 4.0 International license.

### Microbiota composition and formation of metabolites differed between the two fermentation models.

**FIG 2:**
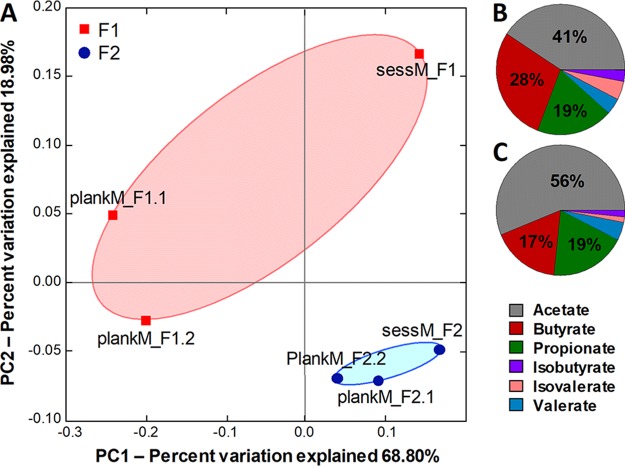
Principal-coordinate analysis (PCoA) and metabolite profile of planktonic and sessile artificial colonic microbiota of independent colonic fermentation models. (A) PCoA plot based on weighted UniFrac distance matrices of planktonic microbiota (plankM) and sessile microbiota (sessM) of fermentation F1 and F2. For better visualization, F1 samples were marked with a red circle and F2 samples with a blue circle. plankM_F1.1 (for cryopreservation) and plankM_F1.2 (for lyophilization) were sampled 21 days apart, whereas plankM_F2.1 (for cryopreservation) and plankM_F2.2 (for lyophilization) were harvested on consecutive days (Fig. S1). SessM_F1 and sessM_F2 were harvested at the end of the fermentation experiments after 48 and 19 days of continuous operation, respectively. (B and C) Metabolite ratios of main SCFA acetate, propionate, and butyrate and BCFA isobutyrate, isovalerate, and valerate of F1 (B) and F2 (C) were calculated from absolute average values determined for three consecutive sampling days. Cryo, cryopreservation; Lyo, lyophilization.

The two independent fermentation models delivered four compositionally and metabolically different microbiotas. The models more consistently maintained the qualitative composition of an individual fecal microbiota in the bead structure than in the planktonic part as indicated by the results from an unweighted UniFrac analysis (Fig. S2). Using principal-coordinate analysis (PCoA), plankM and sessM sequencing data of F1 and F2 were decomposed into principal-component 1 (PC1) and principal-component 2 (PC2), explaining 69% and 19% of the variance, respectively (Fig. 2A). The microbiota samples harvested from F2 were separated from the F1 samples in the PC1 and PC2 directions, indicating that the initial fecal inoculum primarily explained the variation between the intestinal models. Shorter distances were observed between plankM and sessM of F2 (with an average Bray Curtis dissimilarity level of 53%) than between plankM and sessM of F1 (with 63% dissimilarity), suggesting higher similarity between bacterial lifestyles in F2 than in F1.

10.1128/mSystems.00521-19.2 FIG S2 Beta diversity of fecal sample and planktonic and sessile microbiota. Principal-coordinate analysis (PCoA) was performed on the basis of unweighted UniFrac distance matrices of fecal microbiota (FS), planktonic microbiota (plankM), and sessile microbiota (sessM) in fermentation 1 and 2 (F1 and F2). Download FIG S2, TIF file, 0.1 MB. Copyright © 2020 Bircher et al. 2020 Bircher et al. This content is distributed under the terms of the Creative Commons Attribution 4.0 International license.

The metabolic profiles of SCFA, branched-chain fatty acids (BCFA), and fermentation intermediates differed between F1 and F2 (Fig. 2B and C). The effluent harvested from F1 contained major proportions of the SCFA acetate (41%) and butyrate (28%), followed by propionate (19%). Acetate (56%) was the major SCFA in effluents collected from F2, with lower proportions of butyrate (17%) and propionate (19%). BCFA isobutyrate and isovalerate were detected in both fermentation effluents at ratios of 1% to 4%. The levels of fermentation intermediates formate, lactate, and succinate were below the detection limits of the HPLC method, which were 1.5, 3.4, and 1.1 mM, respectively, indicating complete fermentation (Fig. S1).

### Sessile microbiota harbored a unique bacterial and archaeal community distinguishable from planktonic microbiota.

**FIG 3:**
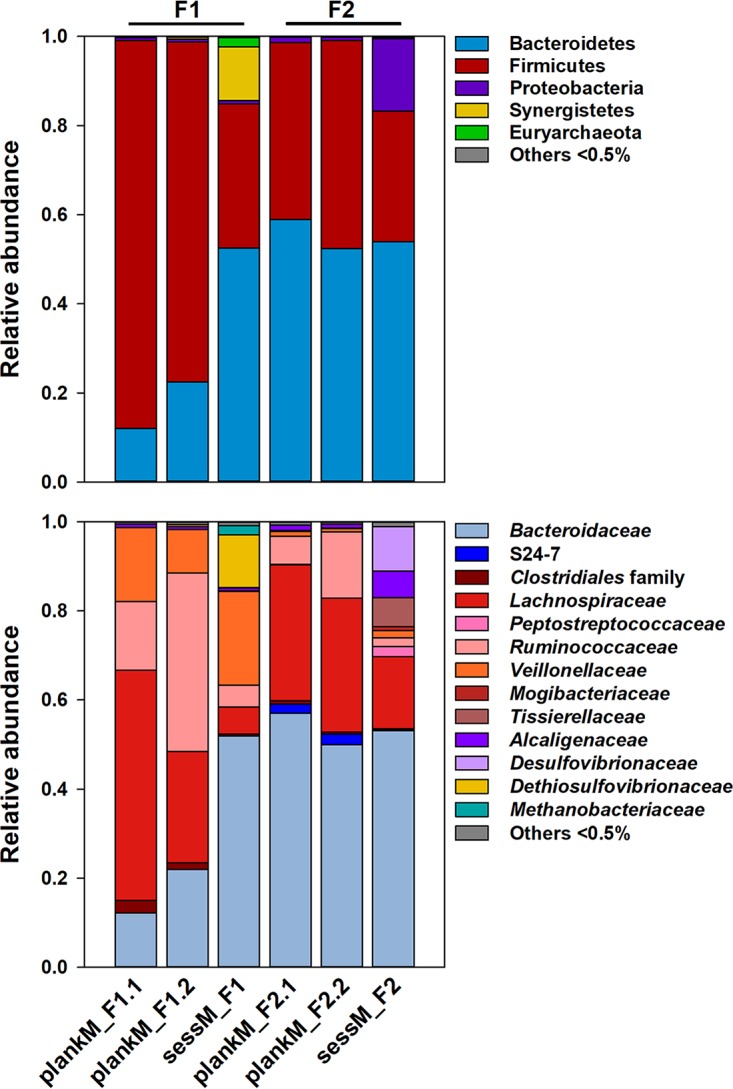
Microbial composition of planktonic and sessile artificial colonic microbiota. Relative levels of abundance of microbial phyla (top panel) and families (bottom panel) of planktonic microbiota (plankM) in F1 and F2 effluents and corresponding sessile microbiota (sessM) immobilized in polymer beads were determined by analysis of the V4 region of the 16S rRNA gene using sequencing. Sampling of plankM for cryopreservation (plankM_F1.1 and plankM_2.1) was conducted 21 days before lyophilization of plankM_F1.2 and 1 day before lyophilization of plankM_F2.2 (Fig. S1).

The immobilized cell intestinal models provided particles for microbes to form sessile mixed-species communities that differed from the planktonic counterpart. The sessM_F1 and sessM_F2 populations harbored higher levels of α-diversity (with 112 and 97 observed species, respectively) than the plankM_F1.1 and plankM_F2.1 populations (with 90 and 85 species present, respectively). At the phylum level, plankM and sessM of both models were mainly represented by *Bacteroidetes* and *Firmicutes* (Fig. 3, top panel). SessM_F1 and sessM_F2 were dominated by *Bacteroidetes*, mainly *Bacteroidaceae* (53% and 59%, respectively), with comparable levels in plankM_F2.1 (54%) but lower abundance in plankM_F1.1 (12%) (Fig. 3, bottom panel). *Firmicutes* were 1.4 to 2.7 times more abundant in plankM than in the corresponding sessM and were mainly represented by the families *Lachnospiraceae* and *Ruminococcaceae* in F2 and additionally by the family *Veillonellaceae* (mainly *Acidaminococcus*) in F1. A lower proportion of *Firmicutes* in sessile microbiota than in planktonic microbiota points toward a lifestyle-associated discriminant of the immobilized cell intestinal model. This is in agreement with other studies from human and rumen samples (5, 7, 9, 35, 36) representing bacterial-lifestyle-impacted microbiota composition.

A comparison of the operational taxonomic units (OTUs) of plankM and sessM produced in the same fermentation model (Fig. S3A and B) showed that a majority of the detected species were ubiquitous in both plankM and sessM. However, 13 species were unique to sessM_F1, and 9 species were found only in sessM_F2 and not in the corresponding plankM_F2. Some of the detected sessile microbiota-specific species, such as Eggerthella lenta, Parabacteroides distasonis, *Rikenellaceae* sp., *Finegoldia* sp., *Bilophila* sp., and Pyramidobacter piscolens, have previously been reported to be associated with colonic biofilms on intestinal biliary stents (37). The relative abundances of lifestyle-specific species were generally below 1%, except for Pyramidobacter piscolens (*Synergistetes*), which contributed 10% of all 16S rRNA gene reads to sessM_F1, and *Bilophila* sp. (*Proteobacteria*), with a relative abundance of 12% in sessM_F2. The occurrence of these two species in the sessile microbiota points at trophic interactions. *Pyramidobacter* sp. and *Bilophila* sp. are phylogenetically different but exhibit similar physiologies. Both genera are asaccharolytic amino acid degraders that mainly produce acetate as well as small amounts of isovalerate, isobutyrate and hydrogen sulfide (38, 39). P. piscolens, rarely cultivated *in vitro*, was successfully isolated from dental plaque communities in coculture with Methanobrevibacter massiliense, suggesting a symbiotic relationship where P. piscolens uses methane produced by M. massiliense to form hydrogen sulfide (40, 41). Indeed, *Methanobrevibacter* sp. was exclusively found in sessM_F1 at a relative abundance of 2% and was the only representative of the archaeal community (Fig. 3, bottom panel). Similarly, Bilophila wadsworthia was previously recovered in mixed cultures, where growth depended on a “helper strain” producing essential growth-promoting factors (quinones) (39, 42). Several bacteria can act as “helpers” supplying quinones, including E. lenta, Eubacterium rectale, and different *Bacteroides* species. Among those bacterial species, here E. lenta was uniquely present in sessM (Fig. S3A and B). Cooperation between microbes involves exchange of metabolites, genetic material, and signaling molecules and is an emergent property of natural biofilms that might also apply to actively immobilized bead communities, allowing growth in microcolonies and promoting cell-cell contacts (18).

10.1128/mSystems.00521-19.3 FIG S3 Venn diagram of microbial composition of planktonic and sessile artificial colonic microbiota. Data represent shared and unique taxa in sessile and planktonic microbiota of F1 (plankM_F1.1 and sessM_F1) (A) and F2 (plankM_F2.1 and sessM_F2) (B). The number inside each square represents the amounts of OTUs observed. Download FIG S3, TIF file, 0.2 MB. Copyright © 2020 Bircher et al. 2020 Bircher et al. This content is distributed under the terms of the Creative Commons Attribution 4.0 International license.

### Planktonic and sessile microbiota showed distinct metabolic profiles and compositional alterations in small-scale batch incubations.

Metabolic activity and growth of fresh plankM and sessM were tested in small-scale batch incubations compared to the subsequently cryopreserved or lyophilized microbiota. We monitored SCFA and intermediate metabolites and BCFA production of fresh unfrozen plankM and sessM processed with cryoprotectants during incubation of strictly anaerobic batches in modified MacFarlane medium over 24 h (fresh plankM or fresh sessM; t_1_) (Fig. 1). We used 16S rRNA gene amplicon sequencing to analyze the microbiota composition and qPCR to determine total bacteria 16S rRNA gene copy numbers. Immediately before incubation, polymer beads harboring the plankM were mechanically homogenized for inoculation.

Fresh plankM and fresh sessM reached similar total bacterial concentrations of log 9.3  ± 0.0 to log 9.5  ± 0.0 16S rRNA gene copies ml^−1^ after 24 h of batch incubation (Table 1). However, sessile lifestyle-associated P. piscolens and *Bilophila* sp. did not establish in batch culture inoculated with resuspended sessile microbiota after bead dissolution (see Table S1 and S2 in the supplemental material). Destroying the bead structure can ultimately lead to destabilization of the symbiotic network and therefore to loss of microbes that can coexist only with another species (43). Similarly, mucosa-associated Lactobacillus mucosae and *Sutterella* spp. (44, 45), present at abundances of 1.6% to 5.2% in sessM_F1 and sessM_F2 (Table S1 and S2), did not grow during batch incubations. Consequently, batch fermentation favors the fraction of resuspended sessile microbiota that can grow in the planktonic state and therefore represents an imperfect activity readout.

**TABLE 1:** Bacterial growth of fresh and stored microbiota^*a*^

Preservation method	Condition	No. of log 16S rRNA gene copies ml^−1^							
		plankM_F1		sessM_F1		plankM_F2		sessM_F2	
		0h	24h	0h	24h	0h	24h	0h	24h
Cryopreservation	Fresh	7.5	9.3 ± 0.1	7.0	9.5 ± 0.0	8.0	9.3 ± 0.1	7.3	9.3 ± 0.0
	9 months	7.5	9.5 ± 0.0	7.2	9.7 ± 0.0	7.9	9.3 ± 0.1	7.5	9.4 ± 0.0

Lyophilization	Fresh	7.5	9.3 ± 0.1	7.1	9.5 ± 0.1	7.8	9.1 ± 0.0	7.2	9.3 ± 0.1
	9 months	7.4	9.3 ± 0.1	6.9	9.5 ± 0.1	7.7	9.5 ± 0.1	7.0	9.2 ± 0.1

a Total numbers of log 16S rRNA gene copies ml*^−^*^1^ after inoculation (0 h) and after 24 h of batch fermentation of planktonic and sessile microbiota (plankM and sessM, respectively) are reported. Data represent means and standard deviations of results from three replicates obtained from fermentation F1 and F2.

10.1128/mSystems.00521-19.5 TABLE S1 Microbial composition of fresh and cryopreserved plankM_F1.1 and sessM_F1 in batch incubation. Relative abundances of microbial genera were determined by analysis of the V4 region by the use of 16S rRNA gene amplicon sequencing. Sampling was conducted immediately after inoculation (0 h) and after 24 h of batch fermentation. OTU, operational taxonomic unit. Download Table S1, DOCX file, 0.02 MB. Copyright © 2020 Bircher et al. 2020 Bircher et al. This content is distributed under the terms of the Creative Commons Attribution 4.0 International license.

10.1128/mSystems.00521-19.6 TABLE S2 Microbial composition of fresh and cryopreserved plankM_F2.1 and sessM_F2 in batch incubation. Relative abundances of microbial genera were determined by analysis of the V4 region by the use of 16S rRNA gene sequencing. Sampling was conducted immediately after inoculation (0 h) and after 24 h of batch fermentation. OTU, operational taxonomic unit. Download Table S2, DOCX file, 0.02 MB. Copyright © 2020 Bircher et al. 2020 Bircher et al. This content is distributed under the terms of the Creative Commons Attribution 4.0 International license.

**FIG 4:**
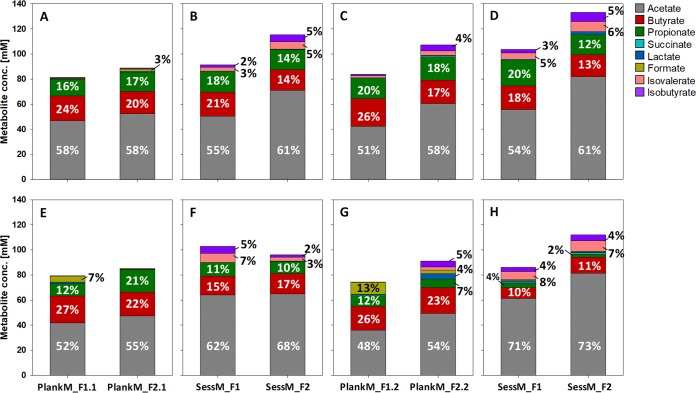
Production of metabolites by fresh, cryopreserved, and lyophilized planktonic and sessile microbiota during batch incubation. Shown are absolute values and ratios of main SCFA, BCFA, and fermentation intermediates produced by fresh plankM_F1.1/1.2/2.1/2.2 (A and C), fresh sessM_F1/2 (B and D), cryopreserved plankM_F1.1/2.1 and sessM_F1/2 (E and F), and lyophilized plankM_F1.2/2.2 and sessM_F1/2 (G and H) after 24 h of batch incubation. Metabolite ratios and absolute concentrations shown represent average values from three independent batch fermentations.

The metabolite data indicate a differentiation in the use of metabolic pathways between sessM and plankM. In general, fresh sessM encompassed a higher degradation capacity than the planktonic counterpart, as indicated by the higher levels of total metabolites formed from degradation of carbohydrates and proteins supplied by the batch medium (Fig. 4A to D). Fresh plankM_F1.1 and plankM_F2.1 formed 80.8  ± 2.0 and 87.7  ± 0.9 mM total SCFA, respectively, while sessM_F1 and sessM_F2 produced 93.0  ± 2.9 and 110.7  ± 1.8 mM SCFA, respectively. Metabolic activity of fresh plankM_F1.2 and plankM_F2.2 confirmed the observed differences (Fig. 4C and D). Higher species diversity, which would provide a greater pool of genes encoding carbohydrate-active enzymes and breakdown of polysaccharides in bacterial cooperation, might be responsible for the increased degradation capacity of sessM compared to the corresponding microbiota in the free-cell state (46–48). In addition, fresh sessM tended to exhibit a more acetogenic character and produced lower portions of butyrate than fresh plankM (Fig. 4A to D). It was previously shown that biofilm communities populating food residues in fecal material produced larger amounts of acetate but lower proportions of butyrate from carbohydrate fermentation than their nonadherent counterparts (48). The BCFA isovalerate and isobutyrate were mainly detected in batch cultures inoculated with fresh sessM_F1 and sessM_F2, amounting to up to 11% of the total metabolites (Fig. 4B and D), but the concentrations were <2.5 mM under conditions of inoculation with plankM_F1 or plankM_F2, except for plankM_F2.2, which produced 4.9  ± 0.4 mM isovalerate and 3.6  ± 0.1 mM isobutyrate (Fig. 4A and C). BCFA are specific end products of protein fermentation by human intestinal microbiota (49, 50). The detection of BCFA indicates either increased proteolytic activity of sessile than planktonic microbiota or faster depletion of available carbohydrate sources due to enhanced metabolic activity forcing the microbes to switch to protein and amino acid metabolism when the carbohydrate energy source is exhausted.

### Effect of cryopreservation and lyophilization on bacterial composition and on the metabolic activity of plankM and sessM after batch reactivation.

To investigate the effects of cryopreservation and lyophilization and of subsequent storage on the composition and activity of the microbiota, plankM and sessM samples from F1 and F2 were added with cryoprotectants (sucrose and inulin [both 5% {wt/vol}] and glycerol [15% {vol/vol}] for cryopreservation and sucrose and inulin [both 5% {wt/vol}] for lyophilization). Samples were either snap-frozen in liquid N_2_ and stored at –80°C for 9 months or lyophilized and stored at 4°C for 9 months before reactivation in small-scale batch fermentations over 24 h (t_2_). As described before, fresh, unfrozen microbiotas processed with cryoprotectants were used as controls (t_1_) (Fig. 1). To determine the degree of compositional similarity between the fresh and the corresponding cryopreserved and lyophilized microbiotas of the same lifestyle, average Bray-Curtis similarity values corresponding to 16S rRNA gene sequencing data were calculated and a principal-component analysis (PCA) was conducted. Main metabolite production, including SCFA, BCFA, and fermentation intermediates, was analyzed using HPLC-RI.

### Overall community structure of stored plankM and sessM was better preserved after cryopreservation than after lyophilization.

Processing and storage did not affect total bacterial numbers in the inocula and after 24 h of batch reactivation of fresh, cryopreserved, and lyophilized plankM and sessM (Table 1).

**FIG 5:**
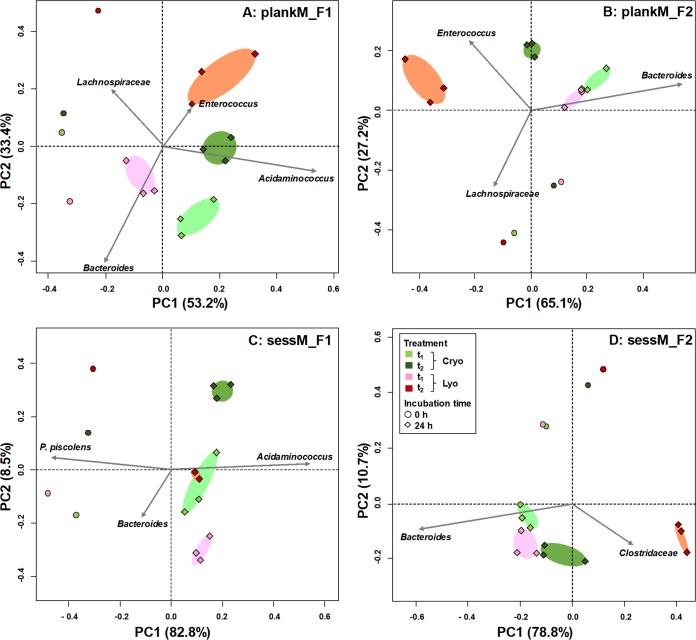
Impact of cryopreservation and lyophilization on microbial community composition. Principal-component analysis (PCA) based on weighted UniFrac distance matrices of fresh (t_1_) and cryopreserved and lyophilized planktonic microbiota (plankM) and sessile microbiota (sessM) stored for 9 months (t_2_) and reactivated in batch fermentation. Each color represents a different treatment. Symbols indicate batch fermentation incubation time (0 or 24 h) of plankM_F1 (A), plankM_F2 (B), sessM_F1 (C), and sessM_F2 (D). Cryo, cryopreservation; Lyo, lyophilization.

Using PCA, genus-level composition data were decomposed into PC1 and PC2, explaining in total between 87% and 92% of the total variance (Fig. 5). PCA showed a stronger effect of lyophilization than cryopreservation, with the results confirmed by average Bray Curtis similarity indices. Cryopreserved microbiotas from F1 and F2 were statistically more similar to the corresponding fresh samples than to the lyophilized microbiotas after 24 h of batch incubation (Table 2). Bray-Curtis index values were highest for cryopreserved plankM_F1.1 samples (0.84  ± 0.03) and lowest for lyophilized sessM_F2 samples (0.33  ± 0.04). Independently of lifestyle, the PCA plots showed that the data representing the fresh samples corresponded to a direction associated with *Bacteroides*. Both cryopreserved and lyophilized plankM separated from fresh samples in a direction positively loaded with *Enterococcus* (Fig. 5A and B). No clear separation between fresh and preserved sample was observed with sessM_F1 (Fig. 5C). Similarly, cryopreserved sessM_F2 closely clustered with fresh samples whereas lyophilized sessM_F2 moved in a direction associated with *Clostridiaceae* (Fig. 5D).

**TABLE 2:** Similarity of fresh and preserved microbiota^*a*^

Preservation method	Life style	Bray-Curtis similarity	
		F1	F2
Cryopreservation	plankM	0.84 ± 0.03	0.78 ± 0.03
	sessM	0.77 ± 0.05^*b*^	0.73 ± 0.06

Lyophilization	plankM	0.75 ± 0.06^*b*^	0.54 ± 0.06^*b*^
	sessM	0.82 ± 0.02	0.33 ± 0.04^*b*^^,^^*c*^

a Bray-Curtis similarity between fresh and preserved planktonic microbiota (plankM) and sessile microbiota (sessM) after 24-h batch fermentations. Data represent means and standard deviations of results from three replicates obtained from fermentation F1 and F2.

b Bray-Curtis similarity of lyophilized microbiota is significantly different from that of cryopreserved microbiota (*P* < 0.05).

c Bray-Curtis similarity of preserved sessM is significantly different from that of preserved plankM (*P* < 0.05).

**FIG 6:**
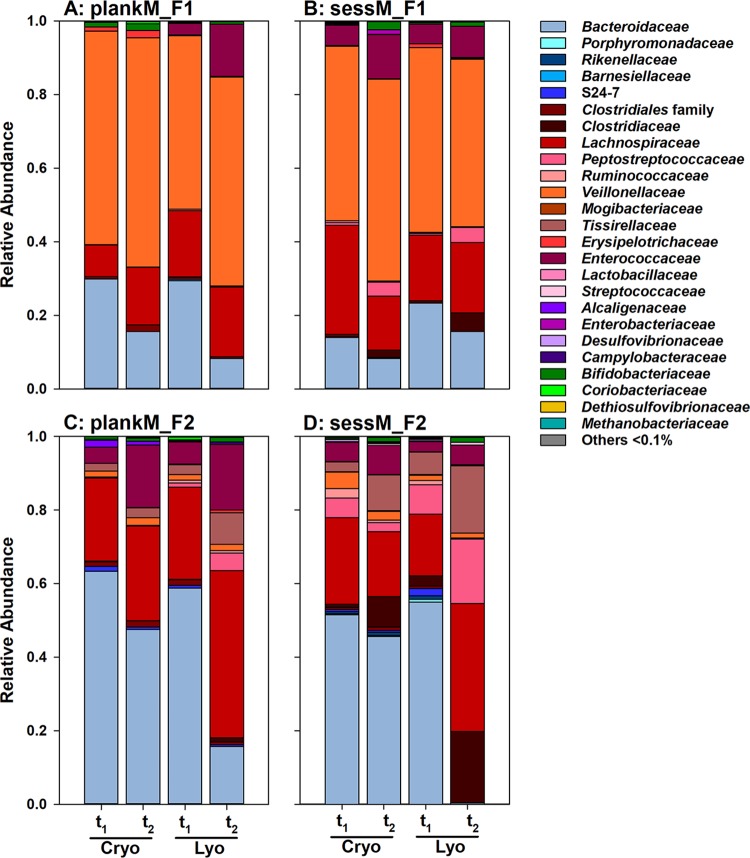
Microbial composition of reactivated fresh and preserved planktonic and sessile microbiota. Relative levels of abundance of bacterial and archaeal families of fresh and preserved planktonic microbiota (plankM) and sessile microbiota (sessM) of fresh (t_1_) and cryopreserved and lyophilized (t_2_) plankM_F1 (A), sessM_F1 (B), plankM_F2 (C), and sessM_F2 (D) were determined by analysis of the V4 region of the 16S rRNA gene amplicon using sequencing after 24 h of batch fermentation. Relative abundance data represent averages of results from three independent batch fermentations. Cryo, cryopreservation; Lyo, lyophilization.

After preservation, we observed a shift in relative abundance from Gram-negative *Bacteroidetes* to Gram-positive *Firmicutes* in both plankM and sessM that was more pronounced in lyophilized microbiotas than in cryopreserved microbiotas (Fig. 6). The relative abundance of *Bacteroidaceae* significantly decreased in reactivated cryopreserved treatments (*P < *0.05) for plankM_F1.1, sessM_F1, and plankM_F2.1 (16%  ± 2%, 8%  ± 1%, and 48%  ± 1%, respectively) compared to fresh microbiota (30%  ± 4%, 14%  ± 3%, and 63%  ± 5%, respectively). The decline in the relative abundance of *Bacteroidaceae* after lyophilization compared to the levels seen with fresh samples was most pronounced in sessM_F2 (from 55%  ± 3% to 0.4  ± 0.2%, *P < *0.05) and least evident in sessM_F1 (from 23%  ± 1% to 19%  ± 6%, *P = *0.12). Lyophilization and cryopreservation can impair growth and can lead to lethal damage for a certain fraction of preserved bacterial cells, clearing niches for more-resistant taxa that are less competitive in fresh microbiota (51). The levels of tolerance of freezing and drying differ greatly between different anaerobic gut microbes (34) and might explain the observed shifts. Previous preservation studies demonstrated that Gram-positive bacteria have an advantage over Gram-negative bacteria in surviving lyophilization (52, 53). It has been suggested that the high portion of cross-linked peptidoglycans in the Gram-positive cell walls provides structural strength during the lyophilization process whereas Gram-negative microbes are more prone to disruption because of their significantly thinner peptidoglycan layer (54). Frozen storage at –80°C similarly affected the microbial compositions of fecal samples by decreasing the *Bacteroidetes*/*Firmicutes* ratio (55).

### *Enterococcaceae* and *Peptostreptococcaceae* benefited from compositional rearrangements after preservation.

Both the *Enterococcaceae* and *Peptostreptococcaceae* families competed more effectively in the complex microbiota after cryopreservation and lyophilization than the members of the *Bacteroidaceae* family (Fig. 6). The relative abundance of *Enterococcaceae* significantly (*P < *0.05) increased from 6%  ± 1%, 4%  ±  0%, and 5%  ± 2% in fresh microbiotas to 12%  ± 0%, 17%  ± 1%, and 8%  ± 2% in cryopreserved sessM_F1, plankM_F2.1, and sessM_F2, respectively, but remained at levels below 0.1% in cryopreserved plankM_F1.1. In lyophilized plankM_F1.2, plankM_F2.2, sessM_F1 and sessM_F2, the relative abundances of *Enterococcaceae* (14%  ± 0%, 18%  ± 5%, 8%, and 5%  ± 1%, respectively) were significantly (*P < *0.05) higher than those seen with fresh microbiota (3%  ± 1%, 6%  ± 0%, 5%  ± 1%, and 3%  ± 0%, respectively). Enterococci are highly adverse conditions, such as undesirable pH, extreme temperature range, or high electrolyte concentration (56). The probiotic Enterococcus faecium (IFA no. 045) previously showed good resistance to lyophilization in the presence of glucose, trehalose, or sucrose, with a survival rate of 80% (57). High viability after preservation might give a competitive advantage for reestablishment under conditions of challenge with a complex microbiota. Similarly, lyophilization enhanced the relative abundance of *Peptostreptococcaceae* in reactivated sessM_F1, plankM_F2.2, and sessM_F2 from 0.5  ± 0.1%, 1%  ± 0%, and 8%  ± 5% in fresh samples to 4%, 5%  ± 2%, and 18% ± 5% in stored samples, respectively. An increase in the relative abundance of *Peptostreptococcaceae* after cryopreservation, from 1%  ± 0% in fresh samples to 4%  ± 1% in lyophilized samples (*P < *0.05), was observed only with sessM_F1.

### Preservation-induced compositional shifts within the order Clostridiales depending on microbiota composition and lifestyle.

Cryopreservation and lyophilization induced major rearrangements within the order *Clostridiales*. A sessile lifestyle favored reestablishment of *Clostridiaceae* after preservation. *Clostridiaceae* were generally present at low (<1%) abundance in all reactivated fresh microbiota and remained at comparable levels in cryopreserved plankM_F1.1 and plankM_F2.1, but the levels increased to 2%  ± 1% in sessM1 and 8%  ± 3% in sessM_F2. Moreover, lyophilization enhanced the abundance of *Clostridiaceae* in sessM (0.1  ± 0.1% to 5% and 8%  ± 4% to 19%  ± 3%, in fresh and lyophilized sessM1_F and sessM_F2, respectively).

Relative abundance shifts of members of *Veillonellaceae* and *Tissierellaceae* were microbiota specific but not lifestyle specific. Members of *Veillonellaceae*, mainly represented by the genus *Acidaminococcus* (Table S1, S3, and S5), were present in plankM_F1.1/1.2 and sessM_F1, while the levels of *Tissierellaceae*, mainly *Peptoniphilus*, differed in plankM_F2.1/2.2 and sessM_F2 (Table S2, S4, and S6). *Veillonellaceae* dominated batch fermentations inoculated with fresh plankM_F1 and sessM_F1 (58%  ± 3% and 47%  ± 7%) and remained at similar levels after cryopreservation (62%  ± 2% and 55%  ± 4%). Lyophilization significantly increased the abundance of *Veillonellaceae* in reactivated plankM_F1 from 47%  ± 3% in fresh samples to 57%  ± 6% in lyophilized samples (*P < *0.05). The relative abundance of *Tissierellaceae* increased from 2%  ± 0% and 3%  ± 0% in fresh samples to 3%  ± 0% and 10%  ± 1% in cryopreserved plankM_F2 and sessM_F2, respectively (*P < *0.05). Lyophilization similarly impacted *Tissierellaceae* in plankM_F2 and sessM_F2, with observed relative abundance levels three times higher in preserved microbiotas (9%  ± 1% and 18%  ± 1%) than in fresh microbiotas (3%  ± 1% and 6%  ± 0%) (*P < *0.05). Lyophilization also specifically enhanced the abundance of *Lachnospiraceae* species within plankM_F2 and sessM_F2, while such an effect was not observed after cryopreservation. The relative abundance of *Lachnospiraceae*, which were already present at 25%  ± 3% and 17%  ± 2% in fresh plankM_F2 and sessM_F2, respectively, almost doubled after reactivation of lyophilized plankM_F2 and sessM_F2 (46%  ± 2% and 35%  ± 4%, respectively) (*P < *0.05).

10.1128/mSystems.00521-19.7 TABLE S3 Microbial composition of fresh and lyophilized plankM_F1.2 in batch incubation. Relative abundances of microbial genera were determined by analysis of the V4 region by the use of 16S rRNA gene sequencing. Sampling was conducted immediately after inoculation (0 h) and after 24 h of batch fermentation. OTU, operational taxonomic unit. Download Table S3, DOCX file, 0.01 MB. Copyright © 2020 Bircher et al. 2020 Bircher et al. This content is distributed under the terms of the Creative Commons Attribution 4.0 International license.

10.1128/mSystems.00521-19.8 TABLE S4 Microbial composition of fresh and lyophilized plankM_F2.2 in batch incubation. Relative abundances of microbial genera were determined by analysis of the V4 region by the use of 16S rRNA gene sequencing. Sampling was conducted immediately after inoculation (0 h) and after 24 h of batch fermentation. OTU, operational taxonomic unit. Download Table S4, DOCX file, 0.01 MB. Copyright © 2020 Bircher et al. 2020 Bircher et al. This content is distributed under the terms of the Creative Commons Attribution 4.0 International license.

10.1128/mSystems.00521-19.9 TABLE S5 Microbial composition of fresh and lyophilized sessM_F1 in batch incubation. Relative abundances of microbial genera were determined by analysis of the V4 region by the use of 16S rRNA gene sequencing. Sampling was conducted immediately after inoculation (0 h) and after 24 h of batch fermentation. OTU, operational taxonomic unit. Download Table S5, DOCX file, 0.01 MB. Copyright © 2020 Bircher et al. 2020 Bircher et al. This content is distributed under the terms of the Creative Commons Attribution 4.0 International license.

10.1128/mSystems.00521-19.10 TABLE S6 Microbial composition of fresh and lyophilized sessM_F2 in batch incubation. Relative abundances of microbial genera were determined by analysis of the V4 region by the use of 16S rRNA gene sequencing. Sampling was conducted immediately after inoculation (0 h) and after 24 h of batch fermentation. OTU, operational taxonomic unit. Download Table S6, DOCX file, 0.01 MB. Copyright © 2020 Bircher et al. 2020 Bircher et al. This content is distributed under the terms of the Creative Commons Attribution 4.0 International license.

### Preservation-induced impairment of metabolic activity was more severe in sessM than plankM.

To evaluate the effect of the environmental stressors of cryopreservation and lyophilization following subsequent storage on microbial activity, we monitored SCFA and BCFA production during 24-h batch fermentations of fresh and preserved plankM and sessM.

Shifts in the metabolic profile seen after frozen storage of cryopreserved microbiota were distinctive for sessM and plankM, indicating a lifestyle-specific effect on SCFA- and BCFA-producing activity (Fig. 4). Compared to fresh conditions, the proportion of acetate produced by cryopreserved sessM_F1 and sessM_F2 was enhanced (from 55% and 61% to 62% and 68%, respectively) at the expense of propionate and butyrate in sessM_F1 and of only propionate in sessM_F2. In contrast, butyrate-producing activity was fully recovered by cryopreserved plankM (105% and 109% of the corresponding fresh plankM_F1.1 and plankM_F2.1 levels) after 24 h of incubation along with slightly lower levels of acetate formation (86% and 91%) (Table 3), shifting the metabolic profile from acetate toward butyrate (Fig. 4E). Accumulation of 6.0  ± 2.0 mM formate in batch fermentation inoculated with cryopreserved plankM1.1 was accompanied by a decrease in the proportion of propionate, from 16% in the metabolic profile of fresh microbiota to 12% after frozen storage (Fig. 6E). Besides affecting final SCFA concentrations, cryopreservation also impacted the reinitiation of metabolic activity. The onset of main SCFA formation by cryopreserved plankM and sessM was significantly delayed compared to that seen with fresh microbiota, while the concentrations of the fermentation intermediates lactate and formate were increased after 6 h of batch fermentation (Fig. S4). The obtained metabolite data point toward a better preservation of the butyrate-producing activity than the propionate-producing activity by the cryopreserved microbiota.

**TABLE 3:** Recovery of metabolic activity after cryopreservation and lyophilization^*a*^

Preservation method	Life style	Metabolite recovery (%)		
		Acetate	Propionate	Butyrate
Cryopreservation	plankM 1	86	73	109
	plankM 2	91	118	105
	sessM 1	126	67	80
	sessM 2	91	59	97

Lyophilization	plankM 1	84	51	87
	plankM 2	81	35	112
	sessM 1	128	19	54
	sessM 2	99	16	72

a Percent metabolite recovery of preserved planktonic microbiota (plankM) and sessile microbiota (sessM) was calculated relative to the average metabolite production of fresh microbiota.

10.1128/mSystems.00521-19.4 FIG S4 Metabolite production by fresh and cryopreserved planktonic and sessile microbiota during batch fermentation. Shown are SCFA, BCFA, and fermentation intermediates produced during batch fermentation of fresh (A to D) and cryopreserved (E to H) planktonic (A, B, E, and F) and sessile (C, D, G, and H) microbiota. Download FIG S4, TIF file, 0.3 MB. Copyright © 2020 Bircher et al. 2020 Bircher et al. This content is distributed under the terms of the Creative Commons Attribution 4.0 International license.

The compositions of butyrate-producing bacteria differed between microbiotas (Fig. 3). The main contributors to butyrate formation during batch fermentations inoculated with fresh plankM_F1 and sessM_F1 were members of the *Lachnospiraceae* family as well as the highly abundant *Acidaminococcus* from the *Veillonellaceae* family. Saccharolytic *Lachnospiraceae* mainly use the butyryl coenzyme A (CoA)/acetate-CoA-transferase pathway for butyrate production, with a net consumption of acetate (58). In contrast, the asaccharolytic Acidaminococcus fermentans and Acidaminococcus intestini species use amino acids to produce acetate and butyrate (59–61). For plankM_F2 and sessM_F2, butyrate may be derived mainly from *Lachnospiraceae* members and *Peptoniphilus* species. *Peptoniphilus* is part of the commensal gut microbiota and includes butyrate-producing, nonsaccharolytic species that use peptone and amino acids as major energy sources (62, 63). Preservation-induced stresses slowed but did not otherwise impair butyrate production. Functional redundancy among the butyrate-producing taxa, the variability in butyrate-producing pathways, and a broad substrate spectrum of plankM and sessM might have maintained butyrate formation after preservation (58, 64).

As observed with the overall community structure, lyophilization also more severely affected metabolic activity than cryopreservation, with stronger detrimental effects on sessM than on plankM (Table 3). Final butyrate concentrations did not significantly differ between fresh and lyophilized plankM, while acetate formation was recovered only partly (84% and 81%, respectively). Propionate production was severely reduced, as shown by recoveries of 51% and 35% by lyophilized plankM_F1.2 and plankM_F2.2, respectively. As a result, metabolic profiles of lyophilized plankM_F1.2/2.2 shifted from acetate and propionate toward butyrate. Production of butyrate and propionate by sessM was impacted to a greater extent by lyophilization than that by plankM, as shown by reduced recovery rates of 54% and 72% butyrate and 19% and 16% propionate by lyophilized sessM_F1 and sessM_F2, respectively. Lyophilization of sessile microbiota was therefore an exception in terms of recovery of butyrate formation. Reduced butyrate production cooccurred with acetate accumulation, likely due to lower net consumption indicative of incomplete fermentation (65). In contrast to butyrate formation, propionate formation was diminished after cryopreservation and, to a greater extent, after lyophilization. Three different bacterial pathways exist that lead to propionate in the human colon; of these, the succinate pathway contributes most to propionate formation (66). Impaired reestablishment of *Bacteroidaceae* after preservation, which employs the succinate pathway, might have led to the reduced propionate formation in plankM and sessM. Similarly to the results seen with cryopreserved plankM_F1.1, formate also accumulated in lyophilized plankM_F1.2 (9.4  ± 0.4 mM) and lactate was still detected after 24 h of incubation of lyophilized plankM_F2 (4.0  ± 2.6 mM) (Fig. 6C). *Enterococcus* species are major lactate producers (67) and might have contributed to the enhanced lactate formation by cryopreserved microbiota that was observed. In turn, increased lactate production can boost cross-feeding and stimulate growth of lactate-utilizing and butyrate-producing bacteria such as Eubacterium hallii and Anaerostipes caccae and therefore indirectly also butyrate formation (68, 69). Indeed, the relative abundance of *Anaerostipes* sp. tended to increase after both lyophilization and cryopreservation. BCFA production activity was maintained in both sessM_F1 and sessM_F2 lyophilized samples compared to control fresh samples (Fig. 4H).

### Conclusion.

This study found that bacterial lifestyle is a determining factor in shaping microbial composition and metabolic capacity of artificially produced colonic microbiota. Sessile microbiotas were metabolically more active than planktonic microbiotas but were also more sensitive to preservation-induced stressors. Our data indicate that cooperating microbes might represent a characteristic component of sessile microbiota possibly affecting compositional stability with respect to freezing and freeze-drying stressors. Accordingly, preservation-induced cell death of a key taxon might provoke a concomitant decrease in the levels of dependent taxa. In general, taxon-dependent individual sensitivities to preservation-induced stressors might lead to a loss of initial community structure but not necessarily to a change in functionality. Functional redundancy, as observed for the butyrate-producing community of both microbiota, might be one trait that guarantees functional stability during exposure to environmental stress. In contrast, if a certain function, such as propionate production, is limited to a small number of taxa, the loss of key taxa can ultimately lead to loss of function. Our data show the feasibility of producing complex artificial microbiotas under conditions of sessile and planktonic lifestyles with the continuous PolyFermS platform inoculated with fecal microbiota immobilized in gel beads and subsequent preservation by freezing and lyophilization. The obtained common results can give first insights into the behavior of planktonic and sessile colonic microbiotas toward preservation-induced stresses; however, for broader assessments of the specific microbiota responses, the experiment should be repeated with a greater number of compositionally different microbiotas. Even though the sessile microbiota exhibited reduced resistance to preservation under our experimental conditions, it may be worthwhile to investigate further the activity of entrapped microbiota directly in bead structures because the resuspension step may have led to a deorganization of the metabolic networks developed during reactor cultivation.

## MATERIALS AND METHODS

### Preparation of protective media.

The applied protective media consisted of 0.1 M phosphate buffer (PB) containing the protective agents sucrose (VWR International AG, Switzerland), inulin (RPN Foodtechnology AG, Switzerland), and glycerol (VWR International AG). Prior to preparation, components of PB and the used protectants were stored in an anaerobic chamber (10% CO_2_, 5% H_2_, 85% N_2_) (Coy Laboratories, USA) overnight to remove residual oxygen. To prepare PB, sodium dihydrogen phosphate (6.0 g liter^−1^) and sodium hydrogen phosphate (7.1 g liter^−1^) (both from Sigma-Aldrich Chemie GmbH, Switzerland) were dissolved in oxygen-free distilled water. The pH was adjusted to 6.8 after the addition of the reducing agents cysteine-HCl and riboflavin (Sigma-Aldrich Chemie GmbH) at final concentrations of 1 g liter^−1^ and 0.3 g liter^−1^, respectively, to counter potential oxygen exposure during processing and storage (28, 70). All three protectants (glycerol [15% {vol/wt}] and sucrose and inulin [both at 5% {wt/vol}]) (GSI) were dissolved in PB for cryopreservation, whereas a protective medium containing only sucrose and inulin (both 5% [wt/vol]) (SI) was used in the lyophilization trials. Protective media were filter sterilized, covered in aluminum foil for protection from light, and stored in an anaerobic chamber before use.

### Preparation of fermentation media.

A nutritive medium for human colonic microbiota was used to investigate metabolic activity and compositional reestablishment of fresh and preserved plankM and sessM in batch fermentation. The medium used for continuous intestinal fermentations was designed to imitate adult ileal chyme entering the colon (71) and was enriched with a filter-sterilized vitamin solution (72). This medium was adapted to conditions in batch fermentation (28) by increasing buffering capacity, reducing carbohydrate content, and adding a SCFA mix (73). Ingredients and preparation were described previously (28).

### Production of artificial colonic microbiota with intestinal fermentation technology.

For each fermentation system, fresh fecal microbiota from a different donor underwent an immobilization procedure. Feces were sampled from two healthy men who were between 30 and 40 years of age and who had not been treated with antibiotics for the previous 3 months. The Ethics Committee of ETH Zürich exempted this study from review because the sample collection procedure had not been performed under conditions of intervention. Informed written consent was obtained from the fecal donors. Immediately after defecation, 5 g fecal material was collected in a preweighted Falcon tube containing 5 ml sterile, prereduced peptone water(Oxoid AG, Switzerland) (0.1%, pH 7). Fecal samples were transported in an anaerobic jar (Anaerojar; Oxoid, England) to maintain anaerobic conditions. All further immobilization steps, which involved encapsulation of fecal microbiota in 1-to-2-mm-diameter polymer gel beads containing gellan (2.5% [vol/wt]), xanthan (0.25% [vol/wt]), and sodium citrate (0.2% [vol/wt]), were performed using an anaerobic chamber as described previously (14). Gellan-xanthan beads (60 ml) were transferred to a glass bioreactor (Sixfors; Ismatec, Switzerland) filled with 140 ml of nutritive medium. Initially, consecutive batch fermentations were carried out to colonize the beads as described previously (28). The system was changed to continuous mode thereafter, with a constant flow rate of 25 ml medium h^−1^, generating a mean retention time of 8 h. The pH was set to 5.7 (controlled by the addition of 2.5 M NaOH), temperature was maintained at 37°C, and stable CO_2_ flow was used to maintain anaerobic conditions in the bioreactors (12). Stability in microbial activity was reached after operation in continuous mode for 10 days, as indicated by stable base consumption and metabolite formation (monitored daily). On the day of the preservation experiments, 1 ml of effluent (plankM) was taken for microbial metabolite analysis by HPLC-RI and microbiota composition analysis by 16S rRNA gene amplicon sequencing. Analysis of sessile microbiota was done on 0.5 g of beads (sessM).

### Harvesting, processing, and preservation of plankM and sessM.

Liquid-phase plankM (25 ml) was directly collected from the bioreactors and transported to an anaerobic chamber, where all further steps were conducted (28). The liquid phase was divided in 12-ml portions in Hungate tubes, and biomass was harvested by centrifugation at 4°C for 10 min at 3,000 × *g*. The pellet was washed in 5 ml PB, centrifuged, and resuspended in 1.2 ml of either SI or GSI medium (10-fold concentration) and was kept for 30 min at room temperature to allow penetration of glycerol. Aliquots of 100 μl either were used immediately for reactivation of fresh plankM and sessM in batch fermentation (see below) or were immersed in liquid nitrogen and either stored at –80°C for 9 months in screw-cap polypropylene cryotubes (Bioswisstec AG, Switzerland) or placed in sterile, prereduced, long stem Vacule cryogenic ampules (Sigma-Aldrich) (Fig. 1) for lyophilization.

For collection of sessM, bioreactors were transferred to an anaerobic chamber and opened to harvest colonized gellan-xanthan beads with a metal sieve after 48 and 19 days of operation of F1 and F2, respectively. Collected beads were washed in PB, and aliquots of 0.5 g were placed into screw-cap polypropylene cryotubes for cryopreservation or in Vacule cryogenic ampules for lyophilization (0.1 g). One hundred microliters of GSI medium was added to the beads in the cryotubes and 50 μl SI medium to the beads in the Vacule cryogenic ampules to completely cover the beads in protective medium, and samples were kept for 30 min at room temperature. Samples were either used immediately for a reactivation test performed under fresh conditions or immersed in liquid nitrogen and stored at 80°C for 9 months or lyophilized.

Lyophilization was conducted with a manifold freeze-dryer (VirTis BenchTop 2K) (SI). Frozen effluent samples in Vacule cryogenic ampules were kept on dry ice before lyophilization to prevent sample melting before the vacuum was reached. Vacule cryogenic ampules were plugged with sterile cotton wool and with blue silica gel as a moisture indicator (Sigma-Aldrich). Samples were freeze-dried for 6 h at 80 mTorr, with a condenser temperature of –80°C. Ampules were flame sealed under vacuum and stored at 4°C for 9 months.

### Reactivation of plankM and sessM in batch fermentation.

To investigate metabolic activity and compositional reestablishment of fresh plankM, three aliquots of GSI-treated or SI-treated plankM were not frozen in liquid nitrogen and were instead immediately reactivated in a small-scale batch fermentation (t_1_). Aliquots were resuspended in 900 μl of anaerobic phosphate-buffered saline (PBS) to the initial volume of the liquid phase.

As with the fresh plankM, three aliquots of GSI-treated or SI-treated sessM were immediately reactivated in batch fermentation without undergoing liquid nitrogen freezing (t_1_). Prior to inoculation, 0.5 g (cryopreservation trial) and 0.1 g (lyophilization trial) portions of fresh beads were mechanically homogenized in 400 and 850 μl PBS containing 0.4% sodium citrate, respectively, by using the tip of a sterile plastic spatula until the beads dissolved.

Serum flasks (50 ml) containing 20 ml anaerobic batch fermentation medium and CO_2_ as headspace gas were inoculated at a concentration of 1% (vol/vol) with each aliquot of plankM and 0.5% (vol/wt) with each aliquot of sessM. Flasks were incubated for 24 h at 37°C under conditions of continuous stirring at 40 rpm. After 0, 4, 6, 8, and 24 h of batch fermentation, 1-ml samples were removed and centrifuged at 13,000 × *g* for 5 min at 4°C. Samples of supernatants for HPLC-IR analysis and microbial pellets for DNA extraction were separately stored at –20°C and –80°C, respectively.

After storage, three aliquots of cryopreserved sessM or plankM were transferred to an anaerobic chamber and quick-thawed at room temperature as described above. Sampling was performed after 0, 4, 6, 8, 12, and 24 h of batch fermentation. Stored lyophilized aliquots (*n* = 3) of plankM and sessM were rehydrated for 1 h in 1 ml and 900 μl PBS, respectively, in an anaerobic chamber. Beads were dissolved as described above. Sampling of batch fermentations was conducted after 0 and 24 h of incubation.

### Metabolite analysis by HPLC-RI.

The metabolic composition of the main SCFA (acetate, propionate, and butyrate), intermediate fermentation metabolites (lactate, succinate, valerate, and formate), and BCFA (isobutyrate and isovalerate) were analyzed in fermentation effluents and batch fermentation samples using HPLC-RI. Supernatants were filtered through a 0.45-μm-pore-size nylon membrane into HPLC vials and closed with crimp caps. Samples were analyzed using an HPLC system (Merck-Hitachi, Germany) equipped with an Aminex HPX-87H column (Bio-Rad) (300 by 7.8 mm) and a refractive index detector (Thermo Fisher Scientific AG). Supernatants (40 μl injection volume) were eluted with 10 mM H_2_SO_4_ at a flow rate of 0.6 ml min^−1^ at 40°C. SCFA, BCFA, lactate, succinate, valerate, and formate were quantified using external standards.

### DNA extraction.

DNA was extracted from 1 ml of fermentation effluent, 0.5 g of beads, and 1 ml of batch fermentation samples using a FastDNA spin kit for soil (MP Biomedicals, France), including mechanical lysis of the cells with a FastPrep instrument (MP Biomedicals, France). DNA was eluted in a final volume of 100 μl. DNA concentration and quality were determined with a NanoDrop ND-1000 spectrometer (Witec AG, Switzerland).

### Quantification of total bacterial numbers.

Total bacterial gene copy numbers were determined by qPCR using the primers Eub_339F (ACTCCTACGGGAGGCAG) and Eub_518R (ATTACCGCGGCTGCTGG) targeting the 16S rRNA gene (74). The qPCR master mix contained 12.5 μl 2× SYBR green Mastermix (Life Technologies, Labgene Scientific Instruments, Switzerland), 0.2 μl of each forward primer and backward primer (5 μM), and 1 μl of genomic DNA in a total volume of 25 μl. The amplification started with a denaturation step at 95°C for 10 min, followed by 40 cycles at 95°C for 15 s and 60°C for 1 min. Melting curve analysis was performed to verify the specificity of amplification. The samples were analyzed in duplicate. Standard curves were generated from 10-fold dilution series (10^2^ to 10^8^ copies) of linearized plasmids containing the target gene.

### Microbiota profiling with 16S rRNA gene amplicon sequencing.

The bacterial composition was determined using a MiSeq platform (Illumina, CA, USA) for tag-encoded 16S rRNA high-throughput sequencing. DNA samples collected after 0 and 24 h of incubation of fresh and preserved sessM and plankM in batch fermentation were selected to assess the reestablishment of bacterial composition. The variable V4 region of the 16S rRNA gene was amplified with the primers nxt_515F (5′-GTGCCAGCMGCCGCGGTAA-3′) and nxt_806_R (5′-GGACTACHVGGGTWTCTAAT-3′). Library preparation and sequencing were conducted in collaboration with the Genetic Diversity Center (GDC) of ETH Zürich, Switzerland. Sequencing was performed using an Illumina MiSeq flow cell with V2 2 × 250-bp paired-end chemistry supplemented with 10% of PhiX.

The raw data sets containing paired-end reads and the corresponding quality scores were merged using settings as previously mentioned (75). The minimum length of the merged reads was 150 bp. The Quantitative Insight Into Microbial Ecology (QIIME) open source software package (1.8.0 and 1.9.0) was used for subsequent analysis steps (76). Purging of the data set from chimeric reads and construction of *de novo* operational taxonomic units (OTU) were performed using the UPARSE pipeline (77). The Green genes database was used as a reference database (78, 79). Alpha and beta diversity analyses were performed using iterative subsampling (18,000 reads/sample) as previously described (80).

### Statistics.

Statistical analysis of metabolite concentration and bacterial abundance data from batch fermentation samples was done using R studio version 3.4.1 (RStudio, Inc., Boston, MA, USA). Data were expressed as means ± standard deviations (SD) of triplicates, except for the lyophilized sessM_F1 data, where only duplicates were available. One lyophilized sessM_F1 data point had to be omitted due to incomplete fermentation of one replicate. A Student's *t* test was performed to compare means of data from cryopreserved and lyophilized samples with those from fresh samples, excluding lyophilized sessM_F1 data due to the limited sample size. Data were tested for normal distribution using the Shapiro-Wilk test and homogeneity of variance using the F-test. Differences were considered significant for α values of ≤0.05.
