# Supplementary figures and images for: Planktonic and Sessile Artificial Colonic Microbiota Harbor Distinct Composition and Reestablish Differently upon Frozen and Freeze-Dried Long-Term Storage
Source: mSystems. 2020 Jan 21;5(1):e00521-19. doi: 10.1128/mSystems.00521-19 (PMC6977070; doi:10.1128/mSystems.00521-19)

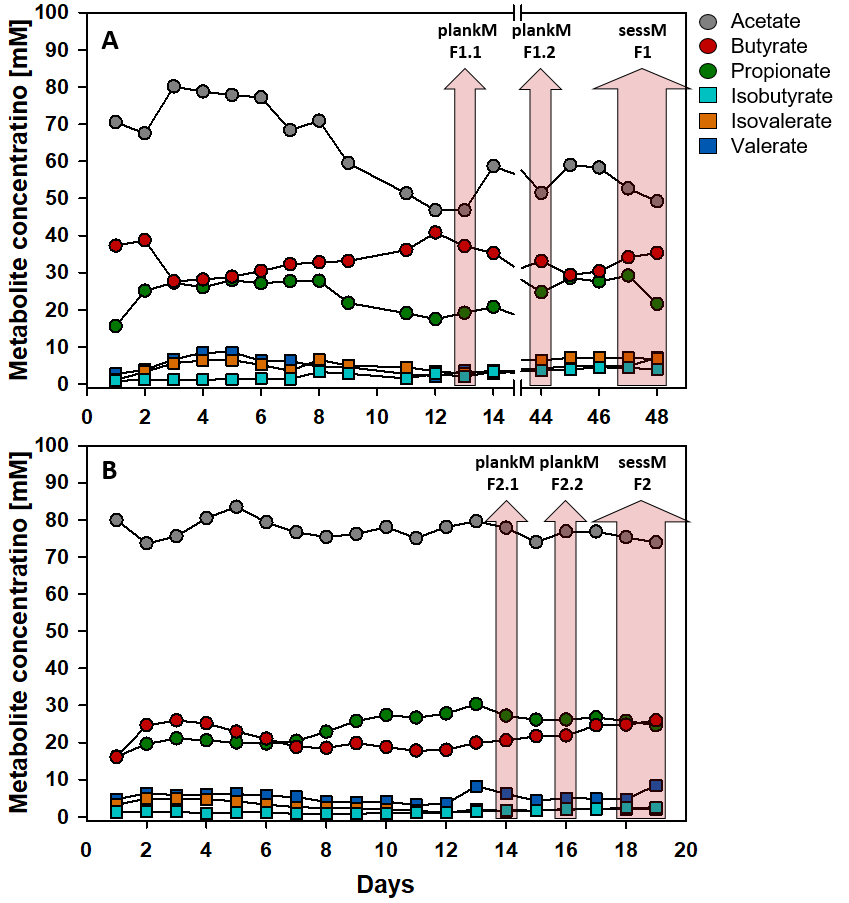

Supplement: FIG S1 [file mSystems.00521-19-sf001.tif]

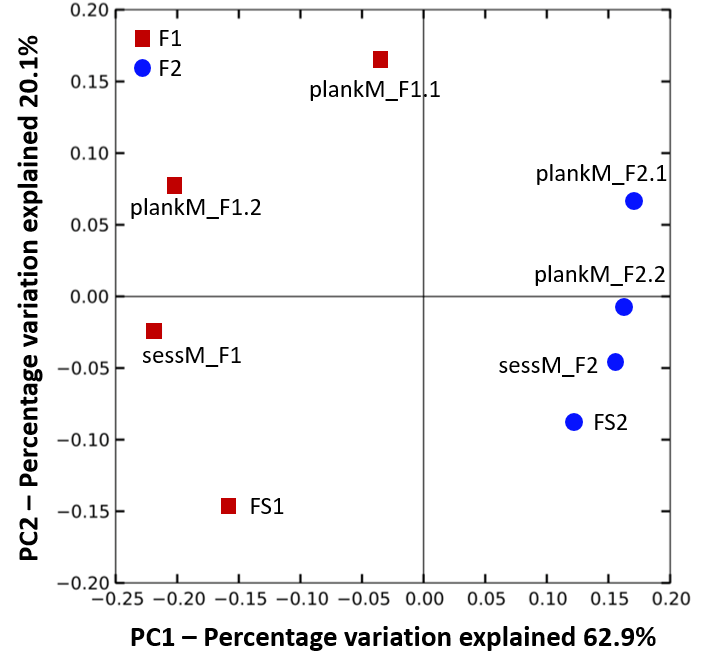

Supplement: FIG S2 [file mSystems.00521-19-sf002.tif]

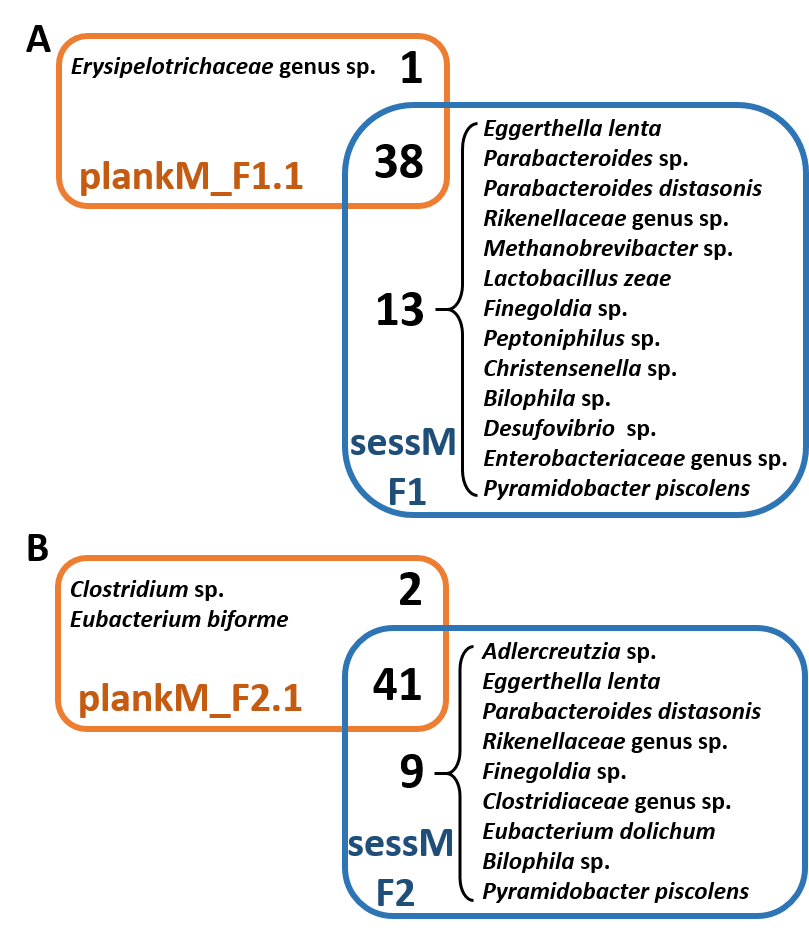

Supplement: FIG S3 [file mSystems.00521-19-sf003.tif]

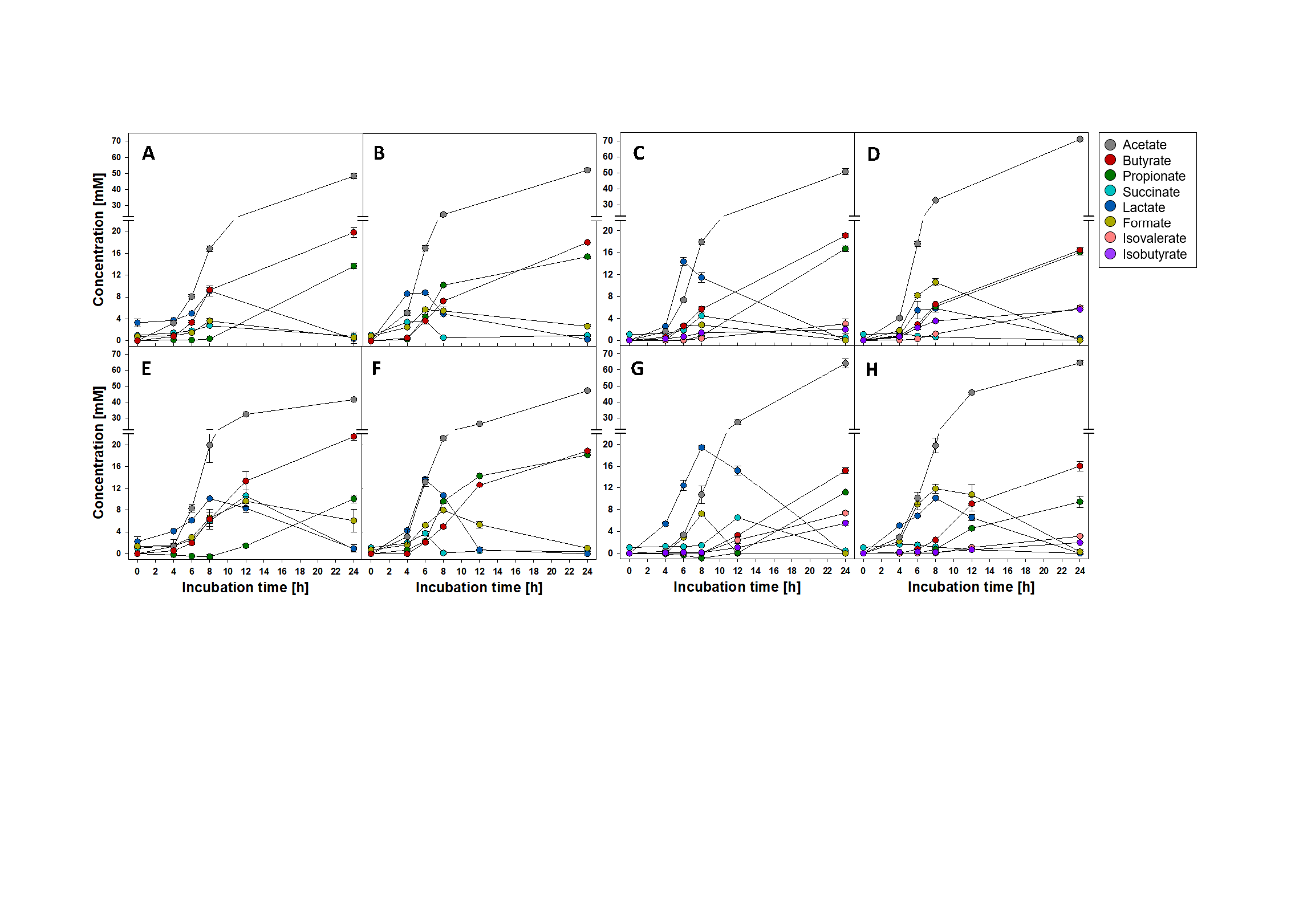

Supplement: FIG S4 [file mSystems.00521-19-sf004.tif]
