# Supplementary material for: Planktonic and Sessile Artificial Colonic Microbiota Harbor Distinct Composition and Reestablish Differently upon Frozen and Freeze-Dried Long-Term Storage
Source: mSystems. 2020 Jan 21;5(1):e00521-19. doi: 10.1128/mSystems.00521-19 (PMC6977070; doi:10.1128/mSystems.00521-19)
Supplement: TABLE S1 [file mSystems.00521-19-st001.docx]

|  | sessM_F1 Fresh | | | sessM_F1 Cryo | | | plankM_F1.1 Fresh | | | plankM_F1.1 Cryo | | |
| --- | --- | --- | --- | --- | --- | --- | --- | --- | --- | --- | --- | --- |
| OTU | 0h | 24h | | 0h | 24h | | 0h | 24h | | 0h | 24h | |
| *Methanobacteriaceae; Methanobrevibacter sp.* | 0.8% | 0.0± | 0.0% | 2.3% | 0.0± | 0.0% | N.D. |  |  | N.D. |  |  |
| *Bifidobacteriaceae; Bifidobacterium adolescentis* | 0.2% | 0.3± | 0.0% | 0.3% | 2.0± | 0.6% | 0.1% | 1.1± | 1.1% | 0.1% | 1.7± | 0.1% |
| *Bacteroidaceae; Bacteroides;Other* | 2.0% | 0.2± | 0.0% | 1.3% | 0.2± | 0.1% | N.D. |  |  | N.D. |  |  |
| *Bacteroidaceae; Bacteroides sp.* | 22.6% | 13.3± | 3.0% | 15.5% | 7.5± | 0.5% | 22.9% | 28.6± | 3.9% | 20.9% | 15.1± | 2.2% |
| *Bacteroidaceae; Bacteroides caccae* | N.D. |  |  | N.D. |  |  | 0.9% | 0.8± | 0.2% | 0.9% | 0.2± | 0.1% |
| *Bacteroidaceae; Bacteroides uniformis* | 3.7% | 0.2± | 0.0% | 2.6% | 0.5± | 0.1% | N.D. |  |  | N.D. |  |  |
| *Enterococcaceae; Enterococcus sp.* | 0.2% | 5.5± | 0.6% | 0.3% | 12.0± | 0.1% | N.D. |  |  | N.D. |  |  |
| *Lactobacillaceae; Lactobacillus mucosae* | 4.1% | 0.0± | 0.0% | 0.6% | 0.0± | 0.0% | 0.7% | 0.0± | 0.0% | 0.2% | 0.0± | 0.0% |
| *Clostridiales; sp.* | 0.7% | 0.6± | 0.0% | 0.6% | 0.2± | 0.1% | 4.8% | 0.7± | 0.3% | 5.6% | 1.8± | 0.5% |
| *Clostridiaceae; sp.* | 0.0% | 0.0± | 0.1% | 0.0% | 2.0± | 0.8% | N.D. |  |  | N.D. |  |  |
| *Lachnospiraceae;Other;Other* | 1.4% | 8.1± | 1.4% | 1.1% | 1.5± | 0.4% | 3.1% | 1.9± | 1.0% | 5.7% | 3.0± | 1.9% |
| *Lachnospiraceae; sp.* | 4.5% | 11.7± | 2.1% | 3.8% | 5.1± | 1.1% | 11.0% | 3.2± | 1.1% | 14.2% | 5.0± | 1.7% |
| *Lachnospiraceae; Anaerostipes sp.* | 0.0% | 1.1± | 0.4% | 0.1% | 1.2± | 0.1% | 0.3% | 0.5± | 0.4% | 0.6% | 4.0± | 1.3% |
| *Lachnospiraceae; Blautia sp.* | 0.3% | 0.8± | 0.1% | 0.3% | 0.8± | 0.2% | 1.5% | 0.3± | 0.1% | 1.7% | 0.5± | 0.2% |
| *Lachnospiraceae; Clostridium hathewayi* | 1.0% | 1.1± | 0.0% | 0.8% | 1.3± | 0.2% | 0.0% | 0.9± | 0.3% | 0.0% | 0.4± | 0.1% |
| *Lachnospiraceae; Coprococcus sp.* | 1.8% | 5.2± | 0.4% | 2.0% | 3.6± | 0.6% | 0.7% | 0.7± | 0.3% | 0.6% | 1.1± | 0.2% |
| *Lachnospiraceae; Dorea sp.* | N.D. |  |  | N.D. |  |  | 0.1% | 0.4± | 0.2% | 0.1% | 0.7± | 0.2% |
| *Lachnospiraceae; Lachnobacterium sp.* | N.D. |  |  | N.D. |  |  | 1.1% | 0.0± | 0.0% | 1.2% | 0.0± | 0.0% |
| *Lachnospiraceae; Lachnospira sp.* | 0.6% | 0.1± | 0.0% | 0.3% | 0.0± | 0.0% | 12.1% | 0.2± | 0.2% | 7.4% | 0.1± | 0.0% |
| *Peptostreptococcaceae; sp.* | 0.0% | 0.8± | 0.4% | 0.0% | 3.8± | 0.9% | N.D. |  |  | N.D. |  |  |
| *Ruminococcaceae; sp.* | 2.0% | 0.2± | 0.0% | 3.3% | 0.2± | 0.1% | 4.9% | 0.0± | 0.0% | 4.1% | 0.0± | 0.0% |
| *Ruminococcaceae; Ruminococcus sp.* | N.D. |  |  | N.D. |  |  | 2.1% | 0.0± | 0.0% | 2.0% | 0.0± | 0.0% |
| *Ruminococcaceae; Faecalibacterium prausnitzii* | 2.3% | 0.0± | 0.0% | 2.0% | 0.0± | 0.0% | 2.4% | 0.0± | 0.0% | 2.8% | 0.1± | 0.0% |
| *Ruminococcaceae; Ruminococcus bromii* | 0.8% | 0.1± | 0.0% | 0.5% | 0.0± | 0.0% | 27.5% | 56.3± | 3.7% | 27.6% | 58.7± | 2.8% |
| *Veillonellaceae; Acidaminococcus sp.* | 16.4% | 45.7± | 6.7% | 22.4% | 52.3± | 4.1% | 0.2% | 1.1± | 0.1% | 0.4% | 1.4± | 0.2% |
| *Veillonellaceae; Dialister sp.* | 6.4% | 1.0± | 0.0% | 10.1% | 1.5± | 0.2% | 0.0% | 0.7± | 0.3% | 0.0% | 2.1± | 0.2% |
| *Veillonellaceae; Mitsuokella sp.* | 0.2% | 0.7± | 0.2% | 0.0% | 1.0± | 0.0% | 0.1% | 1.1± | 0.5% | 0.1% | 2.1± | 0.9% |
| *Erysipelotrichaceae sp.* | N.D. |  |  | N.D. |  |  | N.D. |  |  | N.D. |  |  |
| *Veillonellaceae; Phascolarctobacterium sp.* | 2.6% | 0.0± | 0.0% | 3.2% | 0.0± | 0.0% | 1.2% | 0.0± | 0.0% | 1.4% | 0.0± | 0.0% |
| *Alcaligenaceae; Sutterella sp.* | 0.7% | 0.0± | 0.0% | 0.4% | 0.0± | 0.0% | N.D. |  |  | N.D. |  |  |
| *Enterobacteriaceae; sp.* | 0.0% | 0.2± | 0.1% | 0.0% | 1.3± | 0.1% | N.D. |  |  | N.D. |  |  |
| *Dethiosulfovibrionaceae; Pyramidobacter piscolens* | 21.4% | 0.5± | 0.1% | 23.3% | 0.0± | 0.0% | N.D. |  |  | N.D. |  |  |
