# Supplementary material for: Planktonic and Sessile Artificial Colonic Microbiota Harbor Distinct Composition and Reestablish Differently upon Frozen and Freeze-Dried Long-Term Storage
Source: mSystems. 2020 Jan 21;5(1):e00521-19. doi: 10.1128/mSystems.00521-19 (PMC6977070; doi:10.1128/mSystems.00521-19)
Supplement: TABLE S2 [file mSystems.00521-19-st002.docx]

|  | sessM_F2 Fresh | | | sessM_F2 Cryo | | | plankM_F2.1 Fresh | | | pM_F2.1 Cryo | | |
| --- | --- | --- | --- | --- | --- | --- | --- | --- | --- | --- | --- | --- |
| OTU | 0h | 24h | | 0h | 24h | | 0h | 24h | | 0h | 24h | |
| *Bifidobacteriaceae; Bifidobacterium adolescentis* | 0.2% | 0.4± | 0.1% | 0.8% | 1.2± | 0.1% | N.D. |  |  | N.D. |  |  |
| *Bacteroidaceae; Bacteroides;Other* | 3.6% | 4.1± | 0.3% | 2.9% | 4.7± | 1.5% | 10.9% | 8.5± | 0.9% | 14.3% | 6.6± | 0.3% |
| *Bacteroidaceae; Bacteroides sp.* | 31.3% | 37.3± | 1.2% | 17.7% | 31.2± | 6.4% | 21.9% | 43.2± | 2.3% | 29.5% | 32.0± | 0.1% |
| *Bacteroidaceae; Bacteroides caccae* | 3.5% | 3.9± | 0.1% | 3.0% | 3.0± | 1.3% | 3.8% | 1.1± | 0.1% | 3.2% | 0.4± | 0.1% |
| *Bacteroidaceae; Bacteroides uniformis* | 5.0% | 6.2± | 0.6% | 3.5% | 6.8± | 1.9% | 12.5% | 10.5± | 1.4% | 15.4% | 9.2± | 0.0% |
| *S24-7; sp.* | N.D. |  |  | N.D. |  |  | 1.0% | 1.3± | 0.2% | 0.7% | 0.7± | 0.0% |
| *Enterococcaceae; Enterococcus sp.* | 0.1% | 5.2± | 2.2% | 0.0% | 7.9± | 1.6% | 0.1% | 4.3± | 0.3% | 0.1% | 16.8± | 1.5% |
| *Lactobacillaceae; Lactobacillus mucosae* | 1.6% | 0.0± | 0.0% | 0.2% | 0.0± | 0.0% | N.D. |  |  | N.D. |  |  |
| *Clostridiaceae; sp.* | 0.1% | 0.9± | 0.4% | 0.1% | 8.3± | 3.5% | 0.8% | 1.3± | 0.0% | 1.0% | 1.6± | 0.0% |
| *Lachnospiraceae;Other;Other* | 1.0% | 0.7± | 0.2% | 1.7% | 1.0± | 0.1% | 0.6% | 2.8± | 0.4% | 0.3% | 0.2± | 0.0% |
| *Lachnospiraceae; sp.* | 11.8% | 16.6± | 1.6% | 15.2% | 8.7± | 1.5% | 29.8% | 11.2± | 2.8% | 20.0% | 13.1± | 0.8% |
| *Lachnospiraceae; Anaerostipes sp.* | 0.1% | 0.2± | 0.0% | 0.1% | 3.0± | 3.8% | 0.4% | 0.8± | 0.3% | 0.4% | 3.9± | 0.6% |
| *Lachnospiraceae; Blautia sp.* | 0.9% | 3.2± | 0.1% | 1.9% | 1.1± | 0.3% | 5.6% | 3.9± | 1.2% | 4.8% | 3.0± | 0.2% |
| *Lachnospiraceae; Coprococcus sp.* | 3.1% | 0.6± | 0.1% | 4.0% | 1.6± | 0.3% | N.D. |  |  | N.D. |  |  |
| *Lachnospiraceae; Dorea sp.* | 0.5% | 0.3± | 0.0% | 1.0% | 0.3± | 0.2% | 0.1% | 2.1± | 0.3% | 0.1% | 2.5± | 0.0% |
| *Lachnospiraceae; Dorea formicigenerans* | 3.0% | 0.6± | 0.1% | 4.7% | 0.6± | 0.2% | 1.1% | 0.8± | 0.2% | 0.9% | 1.6± | 0.1% |
| *Peptostreptococcaceae; sp.* | 2.3% | 5.4± | 1.8% | 5.4% | 2.5± | 0.9% | N.D. |  |  | N.D. |  |  |
| *Lachnospiraceae; Lachnospira sp.* | N.D. |  |  | N.D. |  |  | 1.9% | 0.1± | 0.1% | 0.6% | 0.1± | 0.0% |
| *Ruminococcaceae; Faecalibacterium prausnitzii* | 0.8% | 0.3± | 0.0% | 1.0% | 0.0± | 0.0% | 2.3% | 0.0± | 0.0% | 2.4% | 0.0± | 0.0% |
| *Ruminococcaceae; Oscillospira sp.* | 0.8% | 1.8± | 0.1% | 1.2% | 0.5± | 0.1% | N.D. |  |  | N.D. |  |  |
| *Ruminococcaceae; Ruminococcus bromii* | 0.8% | 0.4± | 0.1% | 1.7% | 0.0± | 0.0% | 1.1% | 0.1± | 0.1% | 1.4% | 0.1± | 0.0% |
| *Veillonellaceae; Acidaminococcus sp.* | 0.0% | 2.1± | 0.2% | 0.1% | 0.1± | 0.1% | N.D. |  |  | N.D. |  |  |
| *Veillonellaceae; Dialister sp.* | 1.0% | 0.7± | 0.1% | 1.2% | 0.9± | 0.1% | N.D. |  |  | N.D. |  |  |
| *Veillonellaceae; Phascolarctobacterium sp.* | 0.8% | 1.5± | 0.4% | 1.3% | 1.2± | 0.3% | 2.1% | 0.6± | 0.1% | 1.5% | 1.0± | 0.0% |
| *[Mogibacteriaceae]; sp.* | 1.1% | 0.1± | 0.0% | 3.8% | 0.1± | 0.0% | N.D. |  |  | N.D. |  |  |
| *[Tissierellaceae]; Peptoniphilus sp.* | 9.7% | 2.7± | 0.3% | 13.1% | 9.8± | 0.7% | 0.1% | 2.1± | 0.2% | 0.2% | 2.6± | 0.3% |
| *Erysipelotrichaceae; sp.* | N.D. |  |  | N.D. |  |  | 0.1% | 0.0± | 0.0% | 0.2% | 0.2± | 0.0% |
| *Alcaligenaceae; Sutterella sp.* | 5.2% | 0.0± | 0.0% | 2.7% | 0.0± | 0.0% | 0.3% | 2.0± | 0.1% | 0.8% | 1.1± | 0.2% |
| *Desulfovibrionaceae; Bilophila sp.* | 7.2% | 0.5± | 0.1% | 5.6% | 0.3± | 0.2% | N.D. |  |  | N.D. |  |  |
