# Supplementary material for: Planktonic and Sessile Artificial Colonic Microbiota Harbor Distinct Composition and Reestablish Differently upon Frozen and Freeze-Dried Long-Term Storage
Source: mSystems. 2020 Jan 21;5(1):e00521-19. doi: 10.1128/mSystems.00521-19 (PMC6977070; doi:10.1128/mSystems.00521-19)
Supplement: TABLE S3 [file mSystems.00521-19-st003.docx]

|  | plankM_F1.2 Fresh | | | plankM_F1.2 Lyo | | |
| --- | --- | --- | --- | --- | --- | --- |
| OTU | 0h | 24h | | 0h | 24h | |
| *Bacteroidaceae; Bacteroides; species* | 35.5% | 28.8± | 1.7% | 6.3% | 8.1± | 6.5% |
| *Enterococcaceae; Enterococcus; species* | 0.1% | 3.1± | 1.1% | 0.2% | 14.2± | 0.3% |
| *Lactobacillaceae; Lactobacillus; zeae* | 0.5% | 0.1± | 0.0% | 1.1% | 0.1± | 0.0% |
| *Clostridiales; species* | 0.9% | 0.6± | 0.1% | 1.7% | 0.5± | 0.4% |
| *Lachnospiraceae;Other;Other* | 2.0% | 6.5± | 1.7% | 11.4% | 7.0± | 0.9% |
| *Lachnospiraceae; species* | 11.5% | 8.9± | 2.2% | 28.9% | 8.1± | 1.1% |
| *Lachnospiraceae; Blautia; species* | 1.2% | 0.7± | 0.1% | 3.2% | 0.3± | 0.1% |
| *Lachnospiraceae; Clostridium; hathewayi* | 0.8% | 0.8± | 0.3% | 0.6% | 1.1± | 0.3% |
| *Lachnospiraceae; Coprococcus; species* | 2.9% | 0.3± | 0.1% | 2.4% | 0.5± | 0.3% |
| *Lachnospiraceae; Dorea; formicigenerans* | 0.5% | 0.1± | 0.0% | 1.3% | 0.0± | 0.0% |
| *Lachnospiraceae; Lachnospira; species* | 1.5% | 0.0± | 0.0% | 0.6% | 0.0± | 0.0% |
| *Lachnospiraceae; [Ruminococcus];Other* | 0.7% | 0.1± | 0.0% | 1.6% | 0.5± | 0.6% |
| *Ruminococcaceae; Faecalibacterium; prausnitzii* | 1.9% | 0.0± | 0.0% | 0.4% | 0.0± | 0.0% |
| *Ruminococcaceae; Ruminococcus; species* | 3.0% | 0.0± | 0.0% | 0.8% | 0.0± | 0.0% |
| *Veillonellaceae; Acidaminococcus; species* | 31.8% | 45.1± | 3.2% | 33.9% | 53.9± | 6.2% |
| *Veillonellaceae; Dialister; species* | 0.4% | 1.4± | 0.2% | 1.0% | 1.2± | 0.1% |
| *Veillonellaceae; Mitsuokella; species* | 0.1% | 0.5± | 0.1% | 0.1% | 1.6± | 0.4% |
