# Supplementary material for: Planktonic and Sessile Artificial Colonic Microbiota Harbor Distinct Composition and Reestablish Differently upon Frozen and Freeze-Dried Long-Term Storage
Source: mSystems. 2020 Jan 21;5(1):e00521-19. doi: 10.1128/mSystems.00521-19 (PMC6977070; doi:10.1128/mSystems.00521-19)
Supplement: TABLE S4 [file mSystems.00521-19-st004.docx]

|  | plankM_F2.2 Fresh | | | plankM_F2.2 Lyo | | |
| --- | --- | --- | --- | --- | --- | --- |
| OTU | 0h | 24h | | 0h | 24h | |
| *Bifidobacteriaceae; Bifidobacterium; adolescentis* | 0.0% | 0.3± | 0.1% | 0.0% | 1.3± | 0.2% |
| *Bacteroidaceae; Bacteroides;Other* | 13.2% | 7.0± | 1.7% | 8.6% | 1.9± | 0.6% |
| *Bacteroidaceae; Bacteroides; species* | 32.7% | 41.8± | 1.2% | 21.1% | 8.4± | 2.4% |
| *Bacteroidaceae; Bacteroides; caccae* | 2.8% | 1.5± | 0.4% | 1.8% | 0.2± | 0.1% |
| *Bacteroidaceae; Bacteroides; uniformis* | 13.6% | 10.2± | 1.7% | 10.4% | 2.5± | 0.7% |
| *S24-7; species* | 1.0% | 0.8± | 0.1% | 0.9% | 0.6± | 0.0% |
| *Enterococcaceae; Enterococcus; species* | 0.1% | 6.2± | 0.1% | 0.2% | 19.0± | 7.3% |
| *Clostridiales; species* | 0.6% | 1.5± | 0.2% | 1.0% | 0.7± | 0.4% |
| *Clostridiaceae; species* | 0.0% | 0.1± | 0.0% | 0.0% | 1.2± | 0.2% |
| *Lachnospiraceae;Other;Other* | 0.7% | 1.1± | 0.3% | 1.4% | 10.4± | 0.0% |
| *Lachnospiraceae; species* | 21.3% | 14.5± | 1.4% | 33.7% | 20.2± | 0.9% |
| *Lachnospiraceae; Anaerostipes; species* | 0.4% | 0.8± | 0.0% | 0.9% | 3.1± | 0.6% |
| *Lachnospiraceae; Blautia; species* | 3.0% | 4.3± | 0.2% | 7.4% | 4.5± | 0.2% |
| *Lachnospiraceae; Coprococcus; species* | 1.3% | 0.4± | 0.0% | 1.2% | 0.2± | 0.2% |
| *Lachnospiraceae; Dorea; species* | 0.0% | 1.0± | 0.2% | 0.1% | 1.6± | 0.0% |
| *Lachnospiraceae; Dorea; formicigenerans* | 0.6% | 0.3± | 0.1% | 1.3% | 4.5± | 0.1% |
| *Lachnospiraceae; Lachnospira; species* | 1.4% | 0.2± | 0.0% | 0.4% | 0.1± | 0.0% |
| *Peptostreptococcaceae; species* | 0.0% | 1.2± | 0.2% | 0.0% | 6.1± | 0.7% |
| *Ruminococcaceae; Faecalibacterium; prausnitzii* | 2.2% | 0.0± | 0.0% | 2.0% | 0.0± | 0.0% |
| *Ruminococcaceae; Ruminococcus; bromii* | 1.2% | 0.3± | 0.0% | 1.8% | 0.5± | 0.0% |
| *Veillonellaceae; Phascolarctobacterium; species* | 1.3% | 0.9± | 0.3% | 1.7% | 0.8± | 0.1% |
| *[Tissierellaceae]; Peptoniphilus; species* | 0.1% | 2.2± | 0.1% | 0.4% | 8.1± | 0.5% |
