# Supplementary material for: Planktonic and Sessile Artificial Colonic Microbiota Harbor Distinct Composition and Reestablish Differently upon Frozen and Freeze-Dried Long-Term Storage
Source: mSystems. 2020 Jan 21;5(1):e00521-19. doi: 10.1128/mSystems.00521-19 (PMC6977070; doi:10.1128/mSystems.00521-19)
Supplement: TABLE S5 [file mSystems.00521-19-st005.docx]

|  | sessM_F1 Fresh | | | sessM_F1 Lyo | |
| --- | --- | --- | --- | --- | --- |
| OTU | 0h | 24h | | 0h | 24h |
| *Methanobacteriaceae; Methanobrevibacter; species* | 0.6% | 0.0± | 0.0% | 5.4% | 0.0% |
| *Bifidobacteriaceae; Bifidobacterium; adolescentis* | 0.1% | 0.3± | 0.0% | 0.3% | 1.2% |
| *Bacteroidaceae; Bacteroides;Other* | 1.6% | 0.5± | 0.0% | 1.1% | 0.0% |
| *Bacteroidaceae; Bacteroides; species* | 22.5% | 21.4± | 0.7% | 7.8% | 15.6% |
| *Bacteroidaceae; Bacteroides; uniformis* | 3.2% | 0.7± | 0.1% | 1.5% | 0.0% |
| *Enterococcaceae; Enterococcus; species* | 0.0% | 5.3± | 0.8% | 0.1% | 8.4% |
| *Lactobacillaceae; Lactobacillus; mucosae* | 5.0% | 0.0± | 0.0% | 2.1% | 0.0% |
| *Clostridiaceae; species* | 0.0% | 0.1± | 0.1% | 0.0% | 5.1% |
| *Lachnospiraceae;Other;Other* | 0.8% | 3.8± | 1.1% | 1.5% | 1.5% |
| *Lachnospiraceae; species* | 3.0% | 7.1± | 1.3% | 5.6% | 5.7% |
| *Lachnospiraceae; Anaerostipes; species* | 0.0% | 1.2± | 0.0% | 0.0% | 2.1% |
| *Lachnospiraceae; Blautia; species* | 0.2% | 1.2± | 0.0% | 0.6% | 0.9% |
| *Lachnospiraceae; Clostridium; hathewayi* | 0.4% | 2.2± | 0.1% | 1.0% | 3.9% |
| *Lachnospiraceae; Coprococcus; species* | 1.6% | 1.2± | 0.1% | 2.9% | 1.7% |
| *Lachnospiraceae; Dorea; formicigenerans* | 0.1% | 0.3± | 0.0% | 0.5% | 0.5% |
| *Peptostreptococcaceae; species* | 0.0% | 0.5± | 0.1% | 0.0% | 4.1% |
| *Ruminococcaceae; species* | 2.5% | 0.1± | 0.0% | 4.8% | 0.1% |
| *Ruminococcaceae; Faecalibacterium; prausnitzii* | 2.0% | 0.0± | 0.0% | 3.0% | 0.0% |
| *Veillonellaceae; Acidaminococcus; species* | 13.2% | 48.5± | 3.1% | 21.2% | 44.2% |
| *Veillonellaceae; Dialister; species* | 3.9% | 1.1± | 0.1% | 9.7% | 1.3% |
| *Veillonellaceae; Phascolarctobacterium; species* | 1.7% | 0.0± | 0.0% | 3.9% | 0.0% |
| *Erysipelotrichaceae; species* | 0.0% | 1.1± | 0.3% | 0.0% | 0.2% |
| *Pyramidobacter; piscolens* | 33.2% | 0.3± | 0.1% | 20.6% | 0.0% |
