# Supplementary material for: Planktonic and Sessile Artificial Colonic Microbiota Harbor Distinct Composition and Reestablish Differently upon Frozen and Freeze-Dried Long-Term Storage
Source: mSystems. 2020 Jan 21;5(1):e00521-19. doi: 10.1128/mSystems.00521-19 (PMC6977070; doi:10.1128/mSystems.00521-19)
Supplement: TABLE S6 [file mSystems.00521-19-st006.docx]

|  | sessM_F2 Fresh | | | sessM_F2 Lyo | | |
| --- | --- | --- | --- | --- | --- | --- |
| OTU | 0h | 24h | | 0h | 24h | |
| *Bifidobacteriaceae; Bifidobacterium; adolescentis* | 0.3% | 0.2± | 0.1% | 2.1% | 1.4± | 0.3% |
| *Coriobacteriaceae; species* | 0.2% | 0.0± | 0.0% | 1.2% | 0.0± | 0.0% |
| *Coriobacteriaceae; Adlercreutzia; species* | 0.1% | 0.0± | 0.0% | 1.4% | 0.1± | 0.1% |
| *Bacteroidaceae; Bacteroides;Other* | 3.1% | 4.0± | 0.5% | 1.1% | 0.0± | 0.0% |
| *Bacteroidaceae; Bacteroides; species* | 31.9% | 40.8± | 2.9% | 14.3% | 0.3± | 0.2% |
| *Bacteroidaceae; Bacteroides; caccae* | 4.0% | 4.3± | 0.3% | 2.0% | 0.0± | 0.0% |
| *Bacteroidaceae; Bacteroides; uniformis* | 4.8% | 6.0± | 0.7% | 1.8% | 0.0± | 0.0% |
| *S24-7; species* | 0.4% | 2.0± | 0.2% | 0.4% | 0.0± | 0.0% |
| *Enterococcaceae; Enterococcus; species* | 0.0% | 2.7± | 0.4% | 0.1% | 5.4± | 1.4% |
| *Lactobacillaceae; Lactobacillus; mucosae* | 4.5% | 0.0± | 0.0% | 1.4% | 0.0± | 0.0% |
| *Clostridiaceae; species* | 0.0% | 2.7± | 0.2% | 0.1% | 19.3± | 2.7% |
| *Lachnospiraceae;Other;Other* | 1.1% | 1.3± | 0.4% | 3.0% | 1.2± | 0.0% |
| *Lachnospiraceae; species* | 9.7% | 9.9± | 1.8% | 12.3% | 5.8± | 1.2% |
| *Lachnospiraceae; Anaerostipes; species* | 0.1% | 0.4± | 0.1% | 0.2% | 7.0± | 4.2% |
| *Lachnospiraceae; Blautia; species* | 0.6% | 2.0± | 0.9% | 1.3% | 2.3± | 0.3% |
| *Lachnospiraceae; Coprococcus; species* | 2.7% | 0.8± | 0.4% | 4.8% | 7.6± | 1.5% |
| *Lachnospiraceae; Dorea; species* | 0.4% | 0.3± | 0.1% | 1.2% | 1.8± | 0.6% |
| *Lachnospiraceae; Dorea; formicigenerans* | 3.1% | 0.7± | 0.3% | 6.3% | 2.9± | 1.0% |
| *Lachnospiraceae; [Ruminococcus];Other* | 0.0% | 0.0± | 0.0% | 0.1% | 3.7± | 1.7% |
| *Lachnospiraceae; [Ruminococcus]; species* | 0.1% | 0.1 | 0.0% | 0.1% | 1.4± | 0.7% |
| *Peptostreptococcaceae; species* | 3.0% | 8.0± | 4.8% | 6.7% | 17.5± | 5.1% |
| *Ruminococcaceae; Oscillospira; species* | 1.0% | 0.7± | 0.1% | 1.2% | 0.0± | 0.0% |
| *Ruminococcaceae; Ruminococcus; bromii* | 0.5% | 0.3± | 0.1% | 1.4% | 0.0± | 0.0% |
| *Veillonellaceae; Dialister; species* | 1.5% | 0.8± | 0.3% | 1.6% | 1.2± | 0.3% |
| *Veillonellaceae; Phascolarctobacterium; species* | 1.1% | 0.7± | 0.1% | 1.5% | 0.1± | 0.1% |
| *[Mogibacteriaceae]; species* | 0.9% | 0.2± | 0.0% | 2.5% | 0.0± | 0.0% |
| *[Tissierellaceae]; Peptoniphilus; species* | 6.0% | 6.1± | 0.3% | 15.0% | 18.3± | 0.9% |
| *Alcaligenaceae; Sutterella; species* | 4.9% | 0.0± | 0.0% | 0.8% | 0.0± | 0.0% |
| *Desulfovibrionaceae; Bilophila; species* | 9.7% | 0.4± | 0.0% | 8.1% | 0.0± | 0.0% |
